# Post-translational processing targets functionally diverse proteins in *Mycoplasma hyopneumoniae*

**DOI:** 10.1098/rsob.150210

**Published:** 2016-02-10

**Authors:** Jessica L. Tacchi, Benjamin B. A. Raymond, Paul A. Haynes, Iain J. Berry, Michael Widjaja, Daniel R. Bogema, Lauren K. Woolley, Cheryl Jenkins, F. Chris Minion, Matthew P. Padula, Steven P. Djordjevic

**Affiliations:** 1 The ithree Institute, University of Technology Sydney, PO Box 123, Broadway, New South Wales 2007, Australia; 2 Proteomics Core Facility, University of Technology Sydney, PO Box 123, Broadway, New South Wales 2007, Australia; 3 Department of Chemistry and Biomolecular Sciences, Macquarie University, North Ryde, New South Wales 2109, Australia; 4 NSW Department of Primary Industries, Elizabeth Macarthur Agricultural Institute, Menangle, New South Wales 2568, Australia; 5 School of Biological Sciences, University of Wollongong, Wollongong, New South Wales 2522, Australia; 6 Department of Veterinary Microbiology and Preventative Medicine, Iowa State University, Ames, IA 50011, USA

**Keywords:** two-dimensional gels, adhesins, global proteome, processing, protein-centric

## Abstract

*Mycoplasma hyopneumoniae* is a genome-reduced, cell wall-less, bacterial pathogen with a predicted coding capacity of less than 700 proteins and is one of the smallest self-replicating pathogens. The cell surface of *M. hyopneumoniae* is extensively modified by processing events that target the P97 and P102 adhesin families. Here, we present analyses of the proteome of *M. hyopneumoniae-*type strain J using protein-centric approaches (one- and two-dimensional GeLC–MS/MS) that enabled us to focus on global processing events in this species. While these approaches only identified 52% of the predicted proteome (347 proteins), our analyses identified 35 surface-associated proteins with widely divergent functions that were targets of unusual endoproteolytic processing events, including cell adhesins, lipoproteins and proteins with canonical functions in the cytosol that moonlight on the cell surface. Affinity chromatography assays that separately used heparin, fibronectin, actin and host epithelial cell surface proteins as bait recovered cleavage products derived from these processed proteins, suggesting these fragments interact directly with the bait proteins and display previously unrecognized adhesive functions. We hypothesize that protein processing is underestimated as a post-translational modification in genome-reduced bacteria and prokaryotes more broadly, and represents an important mechanism for creating cell surface protein diversity.

## 1. Background

*Mycoplasma* spp. are bacteria that evolved by a process of degenerative evolution from the low G + C Firmicutes. Mycoplasmas have lost genes for cell wall biosynthesis, and many anabolic processes (including a TCA cycle) are reliant on glycolysis for the production of cellular ATP [1,2]. Mycoplasmas typically have small genomes of less than 1000 kbp and are dependent on the host for the supply of cholesterol for membrane biosynthesis, amino acids, nucleotides and other macromolecular building blocks for cell growth [3]. As such, mycoplasmas are excellent model organisms to examine the complexity of post-translational modifications in prokaryotes.

*Mycoplasma hyopneumoniae* is an agriculturally significant swine respiratory pathogen that causes substantial economic losses, estimated in the billions of dollars per annum [4]. Complete genome sequences of four geographically distinct strains of *M. hyopneumoniae* are available [3,5,6], shedding light on the metabolic capacity, host specialization and evolutionary background of this minimal organism. Genomes range in size from 850 to 920 kb and encode approximately 700 open reading frames (ORFs). The *M. hyopneumoniae* strain 232 genome contains 691 known proteins and 728 annotated genes. A recent proteome analysis of strain 232 identified 8607 unique peptide sequences (false discovery rate of 0.53%) confirming the expression of 70% (483) of the 691 predicted ORFs during culture in Friis broth. This included 171 of the 328 predicted hypothetical proteins (52%), 80% of the lipoprotein genes, and all the P97/P102 adhesin gene families. In the same study, proteogenomic analysis of strain 232 uncovered previously unidentified genes and 5′ extensions to several genes [7]. Transcriptome studies indicate that 92% of predicted ORFs are transcribed in *M. hyopneumoniae* strain 7448 [8]. Seventy-eight non-coding RNAs were also identified in the analysis. Genes with the highest expression levels primarily encoded proteins involved in basal metabolism, as well as chaperones, adhesins, surface proteins, transporters and RNase P. A number of uncharacterized proteins were also identified. The *M. hyopneumoniae* gene encoding the P216 adhesin protein was also presented with a significant number of transcripts (RPKM, reads per kilobase of transcript per million mapped reads: 10 796.4) [8]. While these approaches have shed light on the protein coding capacity of *M. hyopneumoniae*, they do little to understand the extent by which it modifies its proteome post-translationally.

During the early, critical stages of infection, *M. hyopneumoniae* adheres specifically along the entire length of cilia of ciliated epithelial cells that line the trachea, bronchi and bronchioles in the upper respiratory tract of pigs. This association causes ciliostasis, loss of cilia and eventual epithelial cell death, which effectively perturbs mucociliary function. The P97 and P102 adhesin families are central to mediating attachment of *M. hyopneumoniae* to epithelial cilia [9–19]. Notably, all members of the P97 and P102 adhesin families are processed post-translationally to the extent that it is difficult to find evidence of adhesin pre-proteins [9–12,15,17,18,20–23]. Most members of the P97 and P102 families are processed via highly efficient cleavage events typically at S/T–X–F↓–X–D/E sites, but also within stretches of hydrophobic amino acids and by numerous, less efficient cleavage events often in a manner consistent with trypsin-like activity [20–22,24]. Consequently, the surface protein architecture of *M. hyopneumoniae* displays cleavage fragments derived via processing of the P97 and P102 adhesin families by several endopeptidases. What is unclear is how endoproteolysis alters the presentation of surface proteins not related to the P97 and P102 adhesin families, including members of the lipoprotein family.

The current trend in global proteomic analysis has been to use high-speed, ultra-sensitive mass spectrometers combined with orthogonal upfront chromatographic fractionation (i.e. two-dimensional LC–MS/MS) in a peptide-centric manner to characterize proteomes. These high-throughput protocols rely on all proteins in a sample being digested with an efficient protease (e.g. trypsin) into peptides for downstream analysis. Peptide-centric or ‘bottom-up’ approaches are used widely, because peptides are more readily solubilized for fractionation and are amenable to chromatographic separation, and mass spectrometry is more sensitive when analysing peptides, rather than intact proteins [25]. Conversely, protein-centric approaches aim to preserve intact proteins throughout fractionation steps, so that proteoform information may be retained [26], and then discrete proteins or fractions are digested to peptides and analysed individually by mass spectrometry. Protein-centric methods are thus not necessarily ‘top-down’ approaches that aim to analyse individual intact proteins by mass spectrometry [27]. Without selective enrichment, high-throughput peptide-centric approaches can fail to capture post-translational proteolytic modifications and can lead to an oversimplification of the complexity of the proteome. In this study, we applied protein-centric approaches that retain mass context with the aim of identifying proteins that are targets of processing events in *M. hyopneumoniae-*type strain J.

## 2. Experimental procedures

### 2.1. Preparation of *Mycoplasma hyopneumoniae* whole cell lysate

*Mycoplasma hyopneumoniae* (strain J) was grown in modified Friis broth [28] and harvested as described previously [29]. A 0.1 g pellet of *M. hyopneumoniae* cells was resuspended in 7 M urea, 2 M thiourea, 40 mM Tris–HCl pH 8.8, 1% w/v C7BzO and disrupted with four rounds of sonication at 50% power for 30 s bursts on ice. Proteins were reduced and alkylated with 5 mM tributylphosphine and 20 mM acrylamide monomers for 90 min. Insoluble material was pelleted by centrifugation at 16 000*g* for 10 min, and the remaining soluble protein was precipitated in five volumes of ice-cold acetone for 30 min and the pellet air-dried.

For one-dimensional SDS–PAGE, the pellet was resuspended in SDS sample buffer (0.25 M Tris–HCl pH 6.8; 0.25% w/v SDS; 10% glycerol and 0.0025% w/v bromophenol blue). For two-dimensional-PAGE, protein pellets were resuspended in 7 M urea, 2 M thiourea, 1% w/v C7BzO. If solution conductivity was measured to be greater than 200 µS cm^−1^, samples were desalted and buffer exchanged into 7 M urea, 2 M thiourea, 1% w/v C7BzO using a microBioSpin column (Bio-Rad) according to manufacturer's instructions.

### 2.2. TX-114 extraction

*Mycoplasma hyopneumoniae* cell pellets were resuspended in 1% Triton buffer (1% Triton X-114, 10 mM Tris–HCl pH 8.0, 150 mM sodium chloride, 1 mM EDTA) and extracted as previously described [11,30]. The detergent phase sample was mixed with SDS–sample buffer and separated as for GeLC–MS/MS.

### 2.3. Two-dimensional polyacrylamide gel electrophoresis

Two-dimensional gels were run using 250 µg of whole cell lysate with 0.2% pH 3–10 carrier ampholytes (Bio-Rad). Isoelectric focusing was performed using 11 cm pH 4–7 IPG strips (Bio-Rad) and 11 cm pH 6–11 immobiline drystrips (GE Healthcare). Focusing was carried out using a Protean IEF system (Bio-Rad) at a constant 20°C and 50 µA current limit per strip with a three-step programme: slow ramp to 4000 V for 4 h, linear ramp to 10 000 V for 4 h, then 10 000 V until 120 kVh was reached. Following IEF, the strips were equilibrated with 5 ml equilibration solution (2% SDS, 6 M urea, 250 mM Tris–HCl pH 8.5, 0.0025% (w/v) bromophenol blue) for 20 min before the second-dimension SDS–PAGE. The second-dimension gels were run using precast Bio-Rad TGX midi gels with TGS running buffer (Bio-Rad). Reference gels were stained with Coomassie blue G250 overnight and destained with 1% acetic acid to remove background. All visible spots (180 from the pH 4–7 gel and 160 from the pH 6–11 gel) were manually excised from the gel and subjected to in-gel trypsin digestion, before analysis by LC–MS/MS.

### 2.4. One-dimensional gel electrophoresis liquid chromatography tandem mass spectrometry

GeLC–MS/MS was performed on three biological replicates of *M. hyopneumoniae* whole cell lysates, with technical replicates analysed by ion trap MS/MS and Q-TOF MS/MS (representative lane shown in figure 4*a*). One-dimensional GeLC–MS/MS was also performed on a TX-114 detergent fraction and on a high-load lane of whole cell extract (where mass context was not reliably retained owing to macromolecular crowding effects), and these were also analysed by Q-TOF MS.

About 150 µg of protein from any preparation was separated by SDS–PAGE, and fixed and stained with Coomassie blue G-250. Additionally, a high-load lane was run using 500 µg protein from whole cell lysates. Entire gel lanes were cut into 16 equal slices for whole cell lysates, 30 for the high-load lane or 15 for the TX-114 fraction. Gel slices were further diced into approximately 1 mm^2^ cubes, destained, washed and digested in-gel with trypsin for analysis. Identification of proteins was performed following clean-up of peptide fractions using OMIX C18 SPE pipette tips, using one of the LC–MS/MS methods described below.

### 2.5. Expression of recombinant proteins and creation of polyclonal antisera

Expression of recombinant P65 and creation of polyclonal antisera was carried out as described previously [9,14,31].

### 2.6. Blotting

Proteins separated on pH 6–11 two-dimensional gels were transferred to PVDF membranes as described previously [12]. Blots were blocked with 5% (w/v) skim milk powder in PBS with 0.1% Tween 20 (v/v) (PBS-T) at room temperature for 1 h. For detection of immunogenic proteins, membranes were probed with pooled convalescent sera collected from low-health-status *M. hyopneumoniae*-infected pigs described previously [9] diluted 1 : 100 in PBS-T for 1 h, followed by incubation with peroxidase-conjugated anti-pig antibodies diluted 1 : 3000 in PBS-T for 1 h. For detection of adhesin R1 cilium binding domains, membranes were probed with antisera raised against the F3 recombinant fragment that spans the R1 cilium binding domain of MHJ_0194 (F3_P97_); described previously [14] diluted 1 : 100 in PBS-T for 1 h, then peroxidase-conjugated anti-rabbit antibodies diluted 1 : 1500 in PBS-T for 1 h. For detection of P65 fragments, membranes were probed with antisera raised against recombinant P65 diluted 1 : 200 in PBS-T for 1 h, then peroxidase-conjugated anti-rabbit antibodies diluted 1 : 2000 in PBS-T for 1 h. Membranes were washed in three changes of PBS-T between incubations and were developed with SIGMA*FAST* 3,3′-diaminobenzidine tablets (Sigma-Aldrich) as per manufacturer's instructions.

### 2.7. Affinity chromatography for identification of protein interactions

Heparin affinity chromatography and avidin purification of fibronectin-binding proteins and PK15 cell surface protein interactors were performed as described previously [20–22].

Avidin purification of actin- and plasminogen-binding proteins was carried out as follows. Actin from bovine muscle (Sigma-Aldrich) was solubilized in 8 M urea, 20 mM triethylammonium bicarbonate, pH 8.0. Cysteine residues were reduced and alkylated with 5 mM tributylphosphine and 20 mM acrylamide monomers for 90 min at room temperature. Actin monomers were labelled in 20-fold molar excess Sulfo-NHS-LC-Biotin for 3 h at room temperature. Plasminogen from human serum (Sigma-Aldrich) was labelled in 20-fold molar excess Sulfo-NHS-LC-Biotin for 3 h at room temperature. Excess biotin was removed by buffer exchange into PBS using a PD-10 Desalting Column (GE Healthcare, Life Sciences). Biotinylated actin and plasminogen were incubated with avidin agarose (Thermo Scientific) on a rotating wheel for 5 h. The separate slurries were packed into columns and the flow-through collected from each. Unbound ligand was thoroughly washed with PBS. *M. hyopneumoniae* cells were pelleted by centrifugation at 10 000*g* for 20 min, washed with PBS, and gently lysed in 0.5% Triton X-100/PBS. Insoluble material was removed by centrifugation at 16 000*g* for 10 min, and the cleared lysate was incubated with biotinylated ligand–avidin agarose mixtures overnight on a rotating wheel at 4°C. The mixtures were packed into columns, and the unbound proteins were thoroughly washed and collected in PBS. Interacting proteins were eluted with 30% acetonitrile, 0.4% trifluoroacetic acid. The eluting proteins were concentrated using a 3000 Da cut-off filter and acetone precipitated before pelleting by centrifugation. Elutions were subsequently subjected to one-dimensional SDS–PAGE for transfer and detection by blotting or GeLC–MS/MS for protein identification.

Surface proteins were identified by enzymatic cell surface shaving using trypsin for 5 min at 37°C as previously described [12] and cell surface labelling using Sulfo-NHS-LC-Biotin for 30 s at 4°C as previously described [10].

### 2.8. One-dimensional liquid chromatography tandem mass spectrometry using Q-TOF

These methodologies were performed as described previously [21,22]. Briefly, samples were loaded using an Eksigent AS-1 autosampler connected to a Tempo nanoLC system (Eksigent, USA) at 20 µl min^−1^ onto a C8 trap column (Michrom, USA) before washing and elution at 300 nl min^−1^ onto a PicoFrit column (75 µm × 150 mm) packed with Magic C18AQ resin (Michrom, USA). Peptides were eluted and ionized into the source of a QSTAR Elite hybrid quadrupole time-of-flight mass spectrometer (AB Sciex) at 2300 V using the following programmes: 5−50% MS solvent B (98% acetonitrile + 0.2% formic acid) over 30 min for gel slices or 15 min for gel spots, 50−80% MS B over 5 min, 80% MS B for 2 min, 80−5% for 3 min. An intelligent data acquisition experiment was performed, with a mass range of 350−1500 Da scanned for peptides of charge state 2+ to 5+ with an intensity of more than 30 counts scan^−1^. Selected peptides were fragmented, and the product ion fragment masses were measured over a mass range of 50−1500 Da. The mass of the precursor peptide was then excluded for 120 s for gel slices or 15 s for gel spots.

### 2.9. One-dimensional liquid chromatography–mass spectrometry/mass spectrometry using ion trap

Peptide samples were analysed by nanoflow LC–MS/MS (nanoLC-MS/MS) using a LTQ-XL linear ion trap mass spectrometer (Thermo, San Jose, CA), using a fused silica capillary with an integrated electrospray tip (75 µm ID × 70 mm) packed with 100 Å, 5 µm Zorbax C18 resin (Agilent Technologies, CA, USA). An electrospray voltage of 1800 V was applied via a liquid junction upstream of the C18 column. Samples were injected onto the column using a Surveyor autosampler, which was followed by an initial wash step with buffer A (5% v/v acetonitrile, 0.1% v/v formic acid) for 10 min at 1 µl min^−1^. Peptides were eluted from the column with 0–50% buffer B (95% v/v acetonitrile, 0.1% v/v formic acid) for 58 min at 500 nl min^−1^. The column eluate was directed into a nanospray ionization source of the mass spectrometer. Spectra were scanned over the range of 400–1500 amu and, using Xcalibur software (version 2.06, Thermo), automated peak recognition, dynamic exclusion and MS/MS of the top six most intense precursor ions at 35% normalization collision energy were performed.

### 2.10. Mass spectrometry/mass spectrometry data analysis

The MS/MS data files were searched using Mascot (provided by the Australian Proteomics Computational Facility, hosted by the Walter and Eliza Hall Institute for Medical Research Systems Biology Mascot Server) against the LudwigNR database, comprising the UniProt, plasmoDB and Ensembl databases (vQ209. 8785680 sequences, 3087386706 residues), with the following parameter settings. Fixed modifications: none; variable modifications: propionamide, oxidized methionine, deamidated asparagine, n-terminal pyroglutamic acid and carbamoylmethylcysteine cyclization; enzyme: semitrypsin; number of allowed missed cleavages: 3; peptide mass tolerance: 100 ppm or 2.0 Da for data generated by Q-TOF or ion trap instruments, respectively. MS/MS mass tolerance: 0.2 Da or 0.4 Da for data generated by Q-TOF or ion trap instruments, respectively; charge state: 2+ and 3+.

Scaffold v. 3.00.02 (Proteome Software Inc., Portland) was used to validate and compare MS/MS-based peptide and protein identifications. Peptide identifications were accepted if their calculated probability was greater than 95.0% with a false discovery rate of 1.27%, and protein identifications were accepted if their calculated probability using the Peptide Prophet algorithm was greater than 80.0% with a false discovery rate of 2.4%. Protein probabilities were assigned by the Protein Prophet algorithm. Proteins that contained similar peptides and could not be differentiated based on MS/MS analysis alone were grouped to satisfy the principles of parsimony.

The use of multiple techniques improved confidence in ‘one-hit wonders'; proteins identified by a single peptide in a single replicate. Adopting the approach of White *et al*. [32], if the same single peptide was identified in two or more replicates or experiments, the protein was considered to be present, rather than a ‘one-hit wonder’. Similarly, if a single peptide identified a protein in one replicate and a different single peptide identified the same protein in a separate replicate, then the protein was considered to be expressed. Single peptide hits were only retained in the dataset if, after being subjected to manual validation, the MS/MS spectra had a considerable sequence of b- and y-ions that were the dominant ions in the spectra. Six proteins were identified to be true one-hit wonders, with the identifying spectra and fragmentation data shown in electronic supplementary material, figure S4.

### 2.11. *In silico* analyses

Predicted MW and p*I* information for intact proteins and fragments was obtained using ProtParam via ExPASy bioinformatics resource portal (http://web.expasy.org/protparam/) [33]. Transmembrane domain predictions were made using TMPred (http://embnet.vital-it.ch/software/TMPRED_form.html) [34] with default minimum 17 and maximum 33 amino acid length of the hydrophobic portion of the transmembrane helix. The PONDR VSL2 algorithm was used to predict regions of protein disorder (http://pondr.com) [35]. All analyses were performed using sequence data obtained from UniProt (http://www.uniprot.org/) [36].

## 3. Results

### 3.1. Protein-centric approaches to mapping the *Mycoplasma hyopneumoniae* proteome

We applied a series of fractionation technologies that retain mass context to lysates of *M. hyopneumoniae*-type strain J, to determine the diversity of proteins that are targets of endoproteolytic processing. Members of two adhesin families related to P97 and P102 respectively are known to be extensively processed on the cell surface of *M. hyopneumoniae*, but the extent to which proteins on the cell surface are targets of endoproteolytic processing has not been explored. Three hundred and forty-seven unique *M. hyopneumoniae* strain J proteins, representing approximately 52% of the predicted proteome, were identified from the combined experiments following analysis by Scaffold (electronic supplementary material, table S1). Table 1 summarizes the identification of proteins expressed in *M. hyopneumoniae* as detected by each of these methods. Interestingly, two uncharacterized proteins were identified mapping only to strain 232: an 8.8 kDa protein, Q5ZZV3, identified by one peptide in two runs on both ion trap and Q-TOF; and an 11.3 kDa protein, Q5ZZV5, identified by two peptides in one run from ion trap data. A BLAST search of the UniProt database shows that these proteins are conserved among strains 232, 7448 and 168; however, they are not annotated to be present in strain J. Seventy-seven (22%) of the identified proteins are named in UniProt as ‘uncharacterized protein’, despite some sharing homology with proteins that are well characterized in the literature such as P97 and P102 paralogues, MHJ_0369 and MHJ_0368 (Q4A9W4 and Q4A9W5), homologues of Mhp385 and Mhp384 (Q600R9 and Q600S0) respectively, in *M. hyopneumoniae* strain 232 [12].

**Table 1.** Overview of number of identifications by each method.

method	protein IDs	peptide IDs	unique spectra	proteins unique to method
Ge ion trap	331	2774	3832	7
Ge Q-TOF	297	1701	1961	2
Ge high-load	331	1748	2093	6
Ge TX-114	206	846	897	5

**Figure 1.**
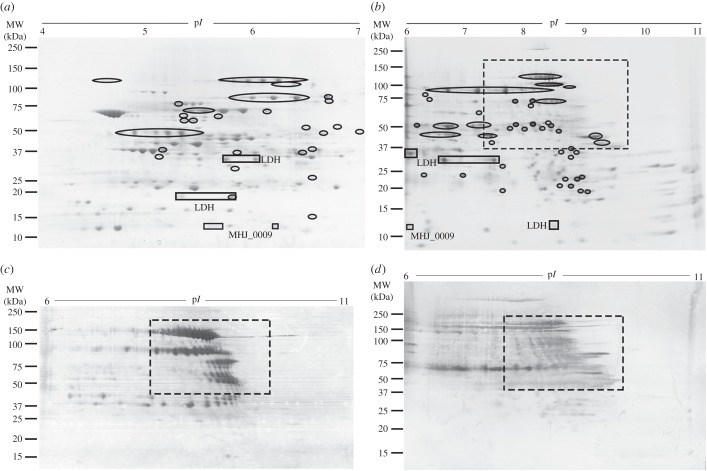
Two-dimensional gels and immunoblots. (*a*) Two-dimensional gel image (pH 4–7) with locations of relevant spots indicated. (*b*) Two-dimensional gel image (pH 6–11) with locations of relevant spots indicated and ‘cloud region’ boxed by a dashed line. Spots identified to contain protein cleavage fragments are circled. Full description of all cut spots and identifications from pH 4–7 and pH 6–11 two-dimensional gels can be found in electronic supplementary material, figures S2 and S3, respectively. (*c*) Two-dimensional blot probed with rabbit serum raised against the F3 recombinant fragment that spans the R1 cilium binding domain of MHJ_0194 (F3_P97_). Strongly staining protein fragments carrying regions of R1 or R1-like fragments of the cilium adhesin P97 and Mhp271 show that proline-rich repeats are highly antigenic. (*d*) A blot probed with pooled convalescent sera from sero-positive pigs. The ‘cloud regions' are also boxed, showing overlap between adhesin fragments and immunoreactive regions of the blots.

GeLC–MS/MS preserves the intact molecular weight of proteins and was a valuable strategy to identify cleavage events that affected the migration of members of the P97 and P102 adhesin families [10,20–22]. Much finer resolution of cleavage fragments was achieved using two-dimensional PAGE. pH 4–7 and 6–11 gels were run using whole cell extracts of *M. hyopneumoniae* (figure 1). Overall, 340 spots comprising 180 spots from a 4–7 isoelectric point gradient gel and 160 spots from a 6–11 isoelectric point gradient gel were resolved well enough to be excised and analysed by LC–MS/MS. Identifications were obtained for 302 spots (159 from p*I* 4–7 and 143 from p*I* 6–11; electronic supplementary material, figures S2 and S3). One hundred and thirty unique proteins were identified from these 302 spots, representing 19% of the predicted proteome (37% of the identifiable proteome).

Eighty-seven proteins were identified from multiple spots. Not all of these, however, could be attributed to processing events, with a significant number of proteins appearing as ‘spot trains' at a specific molecular weight that track along the p*I* gradient. This is likely to be the result of other post-translational modifications that affect p*I*, such as deamidation, or phosphorylation, which has been previously documented in *M. hyopneumoniae* [18]. Of particular interest was the presence of ‘cloud regions' where numerous spots could be detected, but could not be individually resolved (figure 1, boxed). These cloud regions are significant, as similar patterns in the same region have been previously identified when *M. hyopneumoniae* proteins were separated over nonlinear pH 6–11 gels using a different gel system, carried out in a different laboratory and are thus unlikely to be an artefact of sample preparation or gel separation methods [23]. We postulated that these low-abundance cleavage fragments are generated by endoproteolysis of abundantly expressed members of the P97 and P102 adhesin families. A two-dimensional blot probed with rabbit anti-F3_P97_ serum [14] showed that P97, P66 and a range of lower abundance fragments of MHJ_0194 are recognized (figure 1*c*). Identical blots probed with a pool of convalescent sera sourced from pigs testing positive for infection with *M. hyopneumoniae* showed a strong reaction to the low-abundance P97 and P102 adhesin cleavage fragments (figure 1*d*). These observations are consistent with the highly immunoreactive nature of proteins carrying proline-rich repeats [18] such as those recognized by anti-F3_P97_ serum.

### 3.2. The extent of protein processing in *Mycoplasma hyopneumoniae*

Overall, 35 proteins (10% of the identified proteome) showed convincing evidence of proteolytic processing through identification from protein-centric experiments at molecular masses that were not in agreement with the predicted intact mass (table 2; also denoted by asterisks in electronic supplementary material, table S1). Ten of these belong to the P97/P102 adhesin families. Consistent with these data, endoproteolytic processing events have been characterized in P97/P102 adhesin families including MHJ_0194 (P97) [20,23], MHJ_0493 (P97 paralogue P216) [18,22], MHJ_0663 (P97 paralogue P146) [9], MHJ_0369 (P97 paralogue Mhp385) [12], MHJ_264 (P97 paralogue Mhp107) [16], MHJ_0195 (P102) [17,23], MHJ_0494 (P102 paralogue P159/P76/P110) [21,37], MHJ_0662 (P102 paralogue P135/Mhp683) [10], MHJ_0638 (P102 paralogue Mhp384) [12] and MHJ_263 (P102 paralogue P116/Mhp108) [15]. Other proteins showing evidence of cleavage include five uncharacterized proteins, three known surface antigens, two annotated proteases, multiple annotated cytosolic proteins and glycolytic enzymes, such as pyruvate dehydrogenase complex components A, B and D, and lactate dehydrogenase. It is important to note that this list is not exhaustive, as many other proteins were not identified with sufficient sequence coverage to be confirmed as cleavage fragments.

**Table 2.** Cleaved proteins identified from protein-centric analyses. Intact MW shows the calculated mass of the predicted intact protein. Identified masses from one-dimensional GeLC–MS/MS experiments and two-dimensional gels also shown. Putative Fn binding and Heparin binding show mass ranges at which proteins were identified from fibronectin- and heparin-affinity chromatography GeLC–MS/MS experiments, respectively. Masses provided in kDa. D: identified from TX-114 detergent phase GeLC-MS/MS. (N) or (C): identified fragment mapping to N- or C-terminus of the protein. Shading indicates not detected in cell surface analyses. References are provided where proteins have been previously characterized.

	accession	putative cleaved protein ID	gene	intact MW	identified mass	mass from 4 to 7 two-dimensional gels	mass from 6–11 two-dimensional gels	putative Fn binding	heparin binding
adhesins	Q4A925	putative adhesin-like protein P146 [9]	MHJ_0663	147	120–70, 50–40, ∼37–25D	94	35, 38, 45, 92	intact	95–21
	Q4A926	uncharacterized protein [10]	MHJ_0662	135	50–45	52, 74	47, 53, 55, 57	—	57–27, 21–16
	Q4A9J1	putative P76 membrane protein (P159) [21]	MHJ_0494	161	∼110–20	[21]	[21]	∼20	153–27, 21–16
	Q4A9J2	putative P216 surface protein [22]	MHJ_0493	216	∼120, 85–20	[22]	[22]	—	153–27, 21–16
	Q4A9W4	uncharacterized protein [12]	MHJ_0369	114	∼30	25	23, 25	—	95–73, 21–16
	Q4A9W5	putative Lppt protein [12]	MHJ_0368	109	∼50, 30–20D	57	—	—	57–44
	Q4AA66	putative P97-like protein [16]	MHJ_0264	120	∼25	—	100	—	—
	Q4AA67	putative P102-like protein [15]	MHJ_0263	116	∼25, 37, 20	—	19, 41	—	—
	Q4AAD5	uncharacterized protein (P102) [17,23]	MHJ_0195	102	∼60 (N), ∼42 (C)	—	41–46	22–30, 37–70	73–21
	Q4AAD6	uncharacterized protein (P97) [20,23]	MHJ_0194	123	∼120–60, ∼37, 30–20	[20]	[20]	[20]	198–153, 122–57, 27–21, 16–12
surface Ags	P0C0J8	46 kDa surface antigen (p46)	p46 MHJ_0511	46	25–15, >10	44, 47 (multimers)	23, 45	intact	44–27
	Q4A932	putative prolipoprotein p65	MHJ_0656	71	71–50	50, intact	—	intact, 23–37	73–57
	Q4A981	ABC transporter xylose-binding lipoprotein	xylF MHJ_0606	50	∼20D	∼50	—	intact	57–44
annotated cytosolic proteins	Q4A9G1	elongation factor Tu (EfTu)	tuf MHJ_0524	44	44, 21	44	21C	intact, ∼20 C	95–57, 44–27, 21–16
	P0C0J3	l-lactate dehydrogenase (l-LDH) (EC 1.1.1.27) (immunogenic protein p36)	ldh ictD MHJ_0133	34	34–10, ∼20	18, 32, 34	10, 17, 26, 28, 31, 35	intact	34–21, 16–12
	Q4A9I0	acetate kinase (EC 2.7.2.1)	ackA MHJ_0505	44	∼10 (N)	44	43–45	—	44–34
	Q4A9I1	dihydrolipoamide dehydrogenase (EC 1.8.1.4)	pdhD MHJ_0504	66	fragments as multimers	76, 84	—	intact	95–44
	Q4A9P9	3-hexulose-6-phosphate synthase (EC 4.1.2.-)	sgaH MHJ_0436	25	∼15 (C)	23	—	—	44–34
	Q4A9V3	putative thioredoxin	MHJ_0380	13	10 (C)	10	10, 14	—	—
	Q4AAA3	periplasmic sugar-binding protein	rbsB MHJ_0227	44	44–13D	40–44	45	intact	44–34
	Q4AAB1	putative methylmalonate-semialdehyde dehydrogenase (EC 1.2.1.27)	MHJ_0219	54	∼25 (C)	—	51, 53	—	—
	Q4AAL7	pyruvate dehydrogenase (EC 1.2.4.1)	pdhB MHJ_0112	37	37–15	35–40	31, 35	intact	34–27
	Q4AAL8	pyruvate dehydrogenase E1-alpha subunit (EC 1.2.4.1)	pdhA MHJ_0111	42	42–15+multimers	40, 53, 97	45	intact	44–34
	Q4AAL9	adenine phosphoribosyltransferase (APRT) (EC 2.4.2.7)	apt MHJ_0110	19	<10D+multimers	21, 23, 38, 40	31, 35, 45	intact	16–12
	Q4AAR4	chaperone protein DnaK (HSP70)	dnaK MHJ_0063	66	66, <50	47, 68	—	intact, ∼40	73–57, 44–34
	Q4AAR8	glyceraldehyde 3-phosphate dehydrogenase (EC 1.2.1.12)	gap MHJ_0031	37	37-∼10	35, 38	31, 35	intact	—
	Q4AAV7	ATP synthase subunit beta (EC 3.6.3.14)	atpD MHJ_0049	52	20–10D (N)	52	26, 43	—	—
proteases	Q4AAC8	ATP-dependent zinc metalloprotease FtsH (EC 3.4.24.-)	ftsH MHJ_0202	79	79–37	—	—	intact	73–57, 44–34
	Q4A9G3	Oligoendopeptidase F (EC 3.4.24.-)	pepF MHJ_0522	71	∼25, ∼50 15D	—	—	—	73–57
uncharacterized	Q4AA06	uncharacterized protein	MHJ_0326	25	20-<10D	21	—	intact	21–16
	Q4A974	uncharacterized protein	MHJ_0613	95	20–15D (N)	—	100	—	—
	Q4A9G2	uncharacterized protein	MHJ_0523	230	200–75D (C)	—	—	—	—
	Q4A9Q4	uncharacterized protein	MHJ_0431	75	∼25	—	—	—	—
	Q4AAB8	uncharacterized protein	MHJ_0212	236	250, ∼100	191	100 104, 197	—	198–122
	Q4AAU0	uncharacterized protein	MHJ_0009	78	intact, ∼12	—	10 (C)	—	12–10

We selected lipoprotein P65, an uncharacterized protein of unknown function and the cytosolic protein lactate dehydrogenase, which we show are targets of endoproteolytic processing events, to provide an insight into the sequences that are targeted by the processing machinery and present some the putative functions of the cleavage fragments that are generated by these processing events.

### 3.3. Evidence that the P65 lipoprotein is processed on the surface of *Mycoplasma hyopneumoniae*

**Figure 2.**
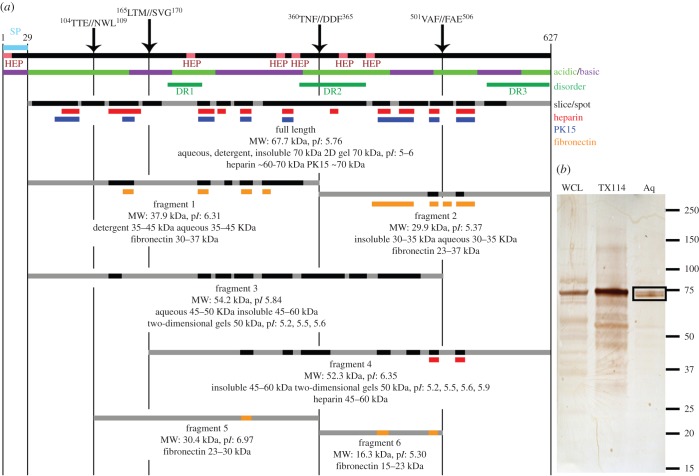
Cleavage map of P65 (MHJ_0656, Q4A932). (*a*) Major features of P65, including a putative signal peptide in light blue (SP), putative heparin binding motifs in light red (HEP), acidic/basic regions and disordered regions with four proposed cleavage sites. Peptides mapping to protein fragments identified from multiple analyses are indicated. Black regions indicate peptides obtained from gel spot or slice data. Peptides identified by affinity chromatography using heparin, PK15 surface proteins and fibronectin-coupled GeLC–MS/MS data are indicated in red, blue and orange, respectively. (*b*) Western blot of *Mycoplasma hyopneumoniae* proteins probed with antisera raised against recombinant P65. The lane labelled WCL contains *M. hyopneumoniae* whole cell lysate. Lanes labelled TX114 and Aq contain biotinylated surface proteins of *M. hyopneumoniae* strain J that partitioned to the detergent and aqueous phases, respectively. Biotinylated proteins were recovered from the Triton and aqueous phases by avidin chromatography prior to gel loading. Multiple cleavage fragments of P65 were detected at masses lower than the abundant intact form. The boxed proteins in the aqueous phase extract at approximately 70 kDa could be attributed to the loss of the lipid anchor in the N-terminus, explaining its abundance in the aqueous phase.

P65, MHJ_0656 (Q4A932), comprises 627 amino acids and encodes a 71 kDa lipolytic lipoprotein with preference for short-chain fatty acids [38]. The N-terminal 29 amino acids comprise the signal sequence and are expected to be removed followed by lipid modification of the cysteine residue at position 30, generating a mature lipoprotein with a mass of 68 kDa and a p*I* of 5.8. We identified P65 as a series of protein spots on a two-dimensional gel with a mass of approximately 68 kDa and a p*I* of 5.8 (figure 2, peptide coverage in black). This 68 kDa molecule was also identified in separate affinity-capture assays using heparin and biotinylated porcine epithelial-like surface proteins as bait (figure 2, peptide coverage in red and blue, respectively). P65 is predicted to display three regions of protein disorder from amino acids 189–228 (DR1), 340–418 (DR2) and 553–627 (DR3) according to the PONDR VSL2 algorithm. One of these, DR1, also overlaps with a coiled coil region (100% probability using the COILS algorithm) between amino acids 214–245, suggesting that this region may not be disordered [39]. Efficient cleavage events are known to occur in S/T–X–F↓X–D/E and related motifs that reside within acidic, disordered regions in the P97 and P102 adhesin families in *M. hyopneumoniae* [9,10,12,17,20–22]. We identified an S/T–X–F↓X–D/E motif in P65 with sequence ^360^T–N–F↓D–D^364^ that resides in DR2, and a cleavage site that cuts at phenylalanine with sequence ^501^V–A–F↓F–A^505^ that is not located within a region of disorder. Both motifs reside within acidic regions that display a p*I* of 5 or less (figure 2). Cleavage at ^360^T–N–F↓D–D^364^ is expected to generate an N-terminal fragment of 38 kDa and a C-terminal fragment of 30 kDa. Tryptic peptides that mapped to the N-terminal 38 kDa (amino acids 30–362) and to the C-terminal 30 kDa regions of P65 (amino acids 365–627) were identified when *M. hyopneumoniae* proteins were enriched by extraction with TX-114 and characterized by LC–MS/MS in gel slices representing proteins with masses between 35–45 and 30–35 kDa, respectively. Cleavage fragments with these masses were also identified by LC–MS/MS during affinity-capture experiments using fibronectin as bait (see fragments 1 and 2 in figure 2). No semi-tryptic peptides were identified to further validate this cleavage site, as lysine resides closely flank this region, making peptide identification following trypsin digestion unlikely. Affinity capture experiments using fibronectin as bait also provided evidence that the 30 kDa C-terminal fragment was cleaved at the ^501^V–A–F↓F–A^505^ site, where cleavage is expected to generate a fragment of 16 kDa. Consistent with this, several tryptic peptides that mapped between amino acids 363–501 were identified in a gel slice containing *M. hyopneumoniae* proteins with masses between 15 and 23 kDa (fragment 6 in figure 2). Protein spots migrating with a mass of approximately 50 kDa on two-dimensional gels produced tryptic peptides mapping to P65, consistent with a fragment that started at position 30 and ended at position 503 (fragment 3 in figure 2), and the semi-tryptic peptide F.^504^FAELNTDQEIK^514^ was identified from gel slices and gel spots by LC–MS/MS, providing further evidence that cleavage occurred at position 503 at the ^501^V–A–F↓F–A^505^ site (table 3; electronic supplementary material, figure S4).

**Table 3.** Semi-tryptic peptides denoting cleavage sites in P65. Site of cleavage that semi-tryptic peptide denotes in P65 according to figure 2, with peptide sequence showing amino acid positions and semi-tryptic terminus and modified amino acids underline. No indicates the number of times a peptide was identified by a given method. In the case where peptides were identified multiple times or in multiple runs, only the highest-scoring peptide is shown.

site	peptide sequence	score	*E*-value	identified	no.
1–1	K.^90^NSLVSYDNLAISGTTTE^106^.N	61	0.0011	peptide-centric^a^	5
1–2	E.^107^NWLYLLNPTK^116^.Y	50	0.011	peptide-centric	1
1–3	E.^107^NWLYLLNPTKYPNGK^121^.M+deamidated (N)	51	0.011	peptide-centric	1
2–1	M.^168^SVGANDPFLAIFNEFK^184^.K	74	4.4 × 10^−6^	WCL ion trap	2
2–2	M.^168^SVGANDPFLAIFNEFK^184^.K	50	0.0025	TX114 Q-TOF	1
2–3	M.^168^SVGANDPFLAIFNEFK^184^.K	82	0.011	gel spots	1
4–1	F.^504^FAELNTDQEIK^514^.E	56	0.0024	WCL Q-TOF	1
4–2	F.^504^FAELNTDQEIK^514^.E	90	0.0014	gel spots	6
4–3	F.^504^FAELNTDQEIK^514^.E+deamidated (Q)	108	2.84 × 10^−5^	gel spots	2

^a^Peptide-centric methods described in electronic supplementary material, figure S4.

Two additional putative cleavage sites were identified in the N-terminal third of P65, ^104^T–T–E↓N–W–L^109^ and ^165^L–T–M↓S–V–G^170^. These sites were identified by the identification of semi-tryptic peptides from GeLC–MS/MS and peptide-centric methods (table 3; electronic supplementary material, figure S4). Complementary C-terminal and N-terminal tryptic peptides identified the cleavage site ^104^T–T–E↓N–W–L^109^, whereas the N-terminal semi-tryptic peptide M.^168^SVGANDPFLAIFNEFK^184^ indicating cleavage at ^165^L–T–M↓S–V–G^170^ was identified from analysis by three different methods (table 3). Cleavage at position 167 is expected to generate two fragments spanning amino acids 30–167 (15.5 kDa; p*I* 4.87) and amino acids 168–627 (52.4 kDa; p*I* 6.35). A fragment with peptide coverage consistent with cleavage at this site was identified by LC–MS/MS in a series of protein spots with mass of approximately 50 kDa and with p*I*s ranging from 5.2 to 5.9. Peptides mapping to the same fragment were also identified from a gel slice containing Triton-X114 insoluble proteins with masses between 45 and 50 kDa, and again following heparin affinity purification, in a gel slice containing proteins with masses between 45 and 60 kDa (fragment 4 in figure 2). We were unable to find a 15.5 kDa protein spanning amino acids 30–167. Cleavage at amino acid position 106 is expected to generate an N-terminal 8.5 kDa protein (p*I* = 4.36) and a C-terminal 59.3 kDa protein (p*I* = 7.00). While neither of these cleavage fragments were identified in our studies, we did identify a single tryptic peptide in a gel slice spanning 30–35 kDa that contained *M. hyopneumoniae* proteins captured during affinity chromatography using fibronectin as bait (fragment 5 in figure 2). This fragment is consistent with cleavage at positions 106 and 362, generating a protein with a mass of 30.4 kDa with a p*I* of 6.97.

### 3.4. Processing events identified in atypical cell surface proteins of *Mycoplasma hyopneumoniae*

**Figure 3.**
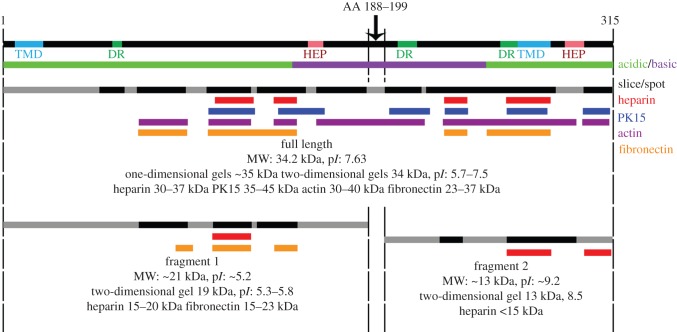
Cleavage map of l-lactate dehydrogenase (LDH; MHJ_0133, P0C0J3). The major features of l-lactate dehydrogenase are shown, including putative transmembrane domains (TMD), putative heparin binding motifs (HEP), putative disordered regions (DR) and acidic/basic regions. A single proposed cleavage site is shown between amino acids 188 and 199, based on peptide coverage. Peptides mapping to protein fragments identified from multiple analyses are indicated. Black regions indicate peptides obtained from gel spot (figure 1) or slice data. Peptides identified by affinity chromatography using heparin, PK15 surface proteins, actin and fibronectin-coupled GeLC–MS/MS data are indicated in red, blue, purple and orange, respectively, at masses as indicated.

Metabolic proteins such as elongation factor Tu, pyruvate dehydrogenase complex components A, B and D, glyceraldehyde-3-phosphate dehydrogenase and l-lactate dehydrogenase (LDH) showed evidence of post-translational processing and were also identified in cell surface analyses (table 2). Evidence that LDH is processed is presented in figure 3. LDH was identified at its predicted mass of 35 kDa and at multiple p*I* between 5.7–7.5 on pH 4–7 and 6–11 two-dimensional gels (figure 3, peptide matches in black). Peptides mapping to LDH were also identified from gel spots at apparent molecular mass of 19 kDa and p*I* 5.3–5.8 on pH 4–7 gels and at 13 kDa and p*I* 8.5 on pH 6–11 gels. The full-length LDH protein was identified in separate affinity-capture assays using heparin, biotinylated fibronectin, actin and porcine epithelial-like surface proteins as bait (figure 3, peptide coverage in red, orange, purple and blue, respectively). While further studies are needed to confirm biologically meaningful interactions between LDH and these host molecules, affinity-capture assays provide independent evidence that regions within LDH bind host molecules and enrich for cleavage fragments.

A single cleavage event between amino acids 188–199 would result in a theoretical N-terminal fragment of approximately 21 kDa with a p*I* of 5.2 and C-terminal fragment of approximately 13 kDa and a p*I* of 9.2, which is similar to the fragments of LDH identified from two-dimensional gels. The shift in p*I* may be attributed to deamidation of asparagine residues at position 121 in the N-terminal fragment and positions 269 and 279 in the C-terminal fragment as detected in peptides identified by GeLC–MS/MS. Neither fragment of LDH was detected from actin or PK15 cell surface binding pulldowns; however, peptides mapping to LDH were identified at masses between 40 and 200 kDa in actin pulldowns, possibly indicating incomplete disassociation from multimeric complexes prior to SDS–PAGE. Peptides mapping to the N-terminal fragment were identified from heparin and fibronectin affinity GeLC–MS/MS experiments in slices at masses 15–20 and 15–23 kDa respectively, whereas peptides mapping to the C-terminal fragment were identified only from heparin affinity GeLC–MS/MS experiments in a slice encompassing masses less than 15 kDa. Although no heparin-binding motif was identified in the C-terminal fragment, the lysine-rich sequence ^290^DKEKEKFAKS^300^ could facilitate interaction with heparin. Further work is needed to determine if ^290^DKEKEKFAKS^300^ binds heparin.

### 3.5. Processing in uncharacterized proteins

**Figure 4.**
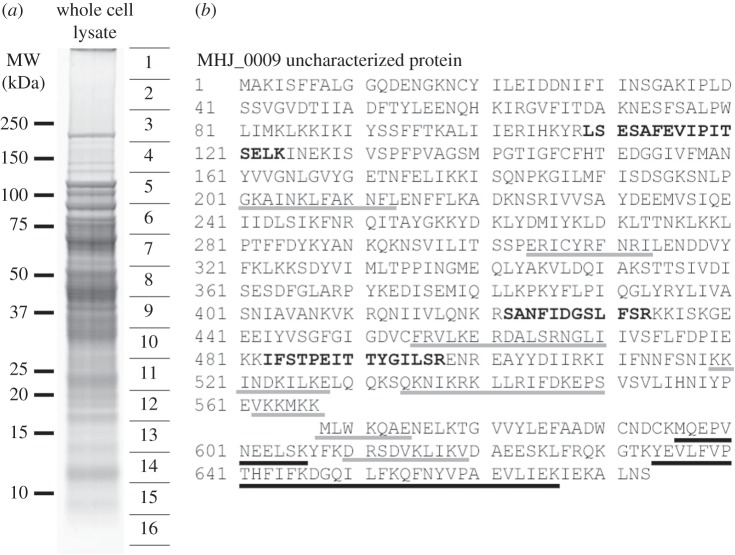
GeLC–MS/MS analysis identified cleavage fragment of MHJ_0009 (Q4AAU0). (*a*) Representative one-dimensional gel of *M. hyopneumoniae* whole cell lysates. The gel lanes were cut into 16 slices (as shown), digested in-gel with trypsin and analysed by LC–MS/MS using ion trap and Q-TOF instruments, allowing protein mass context to be retained. (*b*) Identified peptides mapping to uncharacterized protein MHJ_0009 (Q4AAU0) in bold. Peptides in bold were identified from gel slice 6 at the approximate predicted intact mass (77 kDa). Peptides underlined in black were generated from proteins identified only from slices 13 and 14. Analysis of the C-terminal cleavage fragment spanning amino acids 568–664 with ProtParam indicated that it was 12.5 kDa with a predicted p*I* of 5.47 (see also figure 1). MHJ_0009 was also identified by GeLC–MS/MS from slices at approximately 12 kDa from low-affinity heparin chromatography elutions. Putative heparin binding motifs are underlined in grey.

MHJ_0009 encoding a 77.5 kDa uncharacterized protein (Q4AAU0) was identified consistently in slices 6 (approx. 70–90 kDa), 13 (approx. 13–16 kDa) and 14 (approx. 10–13 kDa) of GeLC–MS/MS using ion trap and Q-TOF analyses from whole cell lysates (figure 4). Peptides identified from replicates of slice 6 mapped to the N-terminus and middle regions of the protein at approximately the predicted mass of the intact protein. Slices 13 and 14, however, are taken from regions of the gel with mass 10–16 kDa, and peptides identified from replicates of these slices mapped only to the C-terminal region of the protein. This C-terminal fragment may represent the product of post-translational proteolytic cleavage. Predicting the true N-terminus of the C-terminal fragment at amino acid position 567 (M) would generate a protein with a mass of 12.5 kDa and p*I* of 5.47 as predicted by ProtParam. We identified the C-terminal fragment from two-dimensional gels at the same approximate molecular mass, with p*I* ranging from approximately 5.5 to 6.2 (figure 1). MHJ_0009 was also identified from GeLC–MS/MS of samples following heparin affinity chromatography from a slice at molecular mass of approximately 10–12 kDa, in elutions carrying proteins with low heparin binding affinity (elution in 150–600 mM NaCl). This is consistent with the presence of putative heparin binding motifs within the C-terminus. Eight putative heparin-binding motifs were identified within MHJ_0009 similar to those described previously [21,22] in both the N- and C-terminal fragments, as denoted by grey underlined regions in figure 4. The protein was identified by the same two C-terminal peptides identified from low molecular mass slices in GeLC–MS/MS (underlined in black in figure 4). The C-terminal fragment of MHJ_0009 contains a thioredoxin-like domain, and a BLAST search of this fragment gives approximately 60% identity to thioredoxin from other *Mycoplasma* species (*M. bovoculi*: E-value: 2 × 10^−43^, score: 375, identity: 62%). Further work is needed to confirm if the C-terminal cleavage fragment displays oxidoreductase activity.

**Figure 5.**
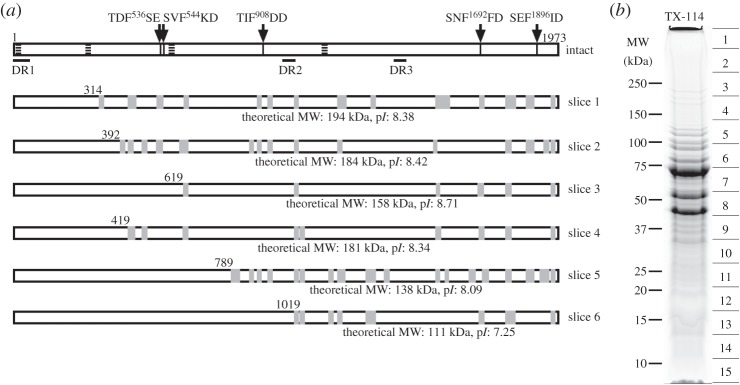
MHJ_0523 cleavage map. (*a*) General features and peptides mapping to MHJ_0523 identified from (*b*) GeLC-MS/MS of a *M. hyopneumoniae* Triton X-114 detergent phase enrichment. Transmembrane domains are indicated by horizontally striped regions and three disordered regions spanning more than 40 amino acids were detected (DR1-3). Five putative S/T-X-F-X-D/E cleavage motifs are indicated by arrows. Peptide coverage identified from individual slices is indicated in grey, and theoretical molecular weight and isoelectric points of protein fragments are shown (according to peptide coverage).

Only one of the cleaved proteins listed in table 2, the uncharacterized protein MHJ_0523, has not also been identified in surfaceome studies using enzymatic shaving and/or cell surface biotinylation [40]. MHJ_0523 encodes a 230 kDa putative lipoprotein and is predicted to possess a transmembrane domain at the N-terminus (TMPred score 1612) and three other putative transmembrane domains (figure 5), which would suggest that the protein is likely to traverse the cell membrane. Extraction of *M. hyopneumoniae* with TX-114 is likely to have concentrated MHJ_0523 into the detergent-soluble fraction, indicating that it may be surface-exposed but expressed at low levels, rendering it undetectable by our shaving/biotin labelling methods. Detection of MHJ_0523 in slice 1 indicates that the molecule is poorly soluble during SDS–PAGE or that it forms large mass multimeric structures. Fragments identified were from the C-terminus ranging from masses upwards of 75 kDa on the TX114 gel, with no coverage of the first 314 amino acids. Five putative S/T–X–F–X–D/E cleavage motifs were identified along the length of the ORF, but we were unable to confirm if processing does occur at these sites.

### 3.6. Proteases identified in *Mycoplasma hyopneumoniae*

Eighteen ORFs have been annotated in the UniProt database (GO annotation) to have putative protease activity, 11 of which have been identified in our study (table 4). Identified proteins with annotated endoprotease activity include MHJ_0522 (Q4A9G3) oligoendopeptidase F, MHJ_0525 (Q4A9G0) Lon protease, MHJ_0636 (Q4A952) tsaD, MHJ_0202 (Q4AAC8) ftsH and MHJ_0568 (Q4A9B9), an uncharacterized protein. These identifications are consistent with those identified in strain 232 [7]. These proteases are likely to carry out the major proteolytic actions that give rise to adhesin fragments, as well as potentially processing other proteins. Lon proteases are bioinformatically predicted to cleave at hydrophobic residues, including phenylalanine (F), and so may play a role in processing at the dominant cleavage motif S/T–X–F↓–X–D/E [9,10]. Additionally, uncharacterized protein MHJ_0568 is predicted to possess a trypsin-like domain, which may be responsible for trypsin-like cleavage events at lysine (K) and arginine (R) residues. Efforts are currently under way to confirm these bioinformatically predicted results.

**Table 4.** Identified proteases of *M. hyopneumoniae*.

accession and locus	identified proteases	surface	gene ontology (GO)
Q4AAC8 MHJ_0202	ATP-dependent zinc metalloprotease FtsH (EC 3.4.24.-)	Y	cell division; integral component of membrane; metalloendopeptidase activity; zinc ion binding
Q4A9G0 MHJ_0525	Lon protease (EC 3.4.21.53) (ATP-dependent protease La)	Y	cellular response to stress; cytoplasm; serine-type endopeptidase activity
Q4AAK4 MHJ_0125	putative aminopeptidase	Y	aminopeptidase activity
Q4A9G3 MHJ_0522	oligoendopeptidase F (EC 3.4.24.-)	Y	metalloendopeptidase activity; zinc ion binding
Q4A9M4 MHJ_0461	leucyl aminopeptidase (EC 3.4.11.1)	Y	aminopeptidase activity; manganese ion binding; metalloexopeptidase activity
Q4A929 MHJ_0659	XAA-PRO aminopeptidase (EC 3.4.11.9)	Y	aminopeptidase activity; metalloexopeptidase activity
Q4AAM9 MHJ_0098	ATP-dependent protease binding protein	N	ATP binding; nucleoside-triphosphatase activity; peptidase activity
Q4A952 MHJ_0636	tRNA N6-adenosine threonylcar-bamoyltransferase (EC 2.6.99.4)	N	cytoplasm; iron ion binding; metalloendopeptidase activity
Q4AAG1 MHJ_0169	methionine aminopeptidase (MAP) (MetAP) (EC 3.4.11.18)	N	metal ion binding; metalloaminopeptidase activity; protein initiator methionine removal
Q4AAS7^a^ MHJ_0022	signal peptidase I (EC 3.4.21.89)	N	integral component of membrane; serine-type peptidase activity
Q4A9B9 MHJ_0568	uncharacterized protein	N	serine-type endopeptidase activity

^a^Although MHJ_0022 has a signal peptidase I signature motif, existing biochemical data from amino-terminal sequence analysis of amino-terminal cleavage products indicates that this species lacks SPase I activity. ‘Surface’ indicates proteins were (Y) or were not (N) identified in cell surface shaving or biotinylation experiments.

## 4. Discussion

### 4.1. Protein-centric approaches to mapping the *Mycoplasma hyopneumoniae* proteome

While our protein-centric separation approaches identified only 347 proteins, representing 52% of the *M. hyopneumoniae* proteome [32,41–44], they enabled us to characterize endoproteolytic processing events in 35 functionally diverse, surface-associated proteins. The unidentified portion of the proteome consisted of 198 uncharacterized ORFs, which may be of low abundance or have a high rate of turnover, or may not be transcribed under the growth conditions used in our analyses. Additionally, some ORF sequences that remain unidentified contain too many (or rarely too few) lysine and/or arginine residues, making the tryptic peptides generated by digestion undetectable by the methods used. Instrument sensitivity only partially explains why we did not identify a greater proportion of the proteome, given that we were only able to identify 70% (483) of the 691 predicted ORFs in strain 232 during culture in Friis broth [7]. Our approach is consistent with our primary goal to preserve mass-context prior to mass spectrometry as a means to identify the gamut of proteins targeted by processing mechanisms. Two-dimensional PAGE was able to resolve individual proteins and isoforms, providing information about post-translational modifications, whereas one-dimensional GeLC–MS/MS methods are higher-throughput, making them better suited for global proteome identification. The protein-centric approaches used in our studies provided insights into the extent of protein processing in *M. hyopneumoniae*. Table 2 lists the proteins cleaved in *M. hyopneumoniae*. Notably, almost all of the proteins in table 2 were identified in a comprehensive surfaceome analysis conducted using cell shaving and surface biotinylation methodologies (J.L.T., B.B.A.R. & S.P.D. 2012 unpublished data). Our data suggest that protein processing is a post-translational modification that occurs with greater frequency than is currently recognized and occurs in a wide range of functionally diverse cell surface proteins. This is consistent with the processing machinery being associated with the cell surface or with the general secretory pathway. On a cautionary note, it remains to be determined what mechanisms are needed to export proteins with canonical functions in the cytosol onto the cell surface [45–47]. Nonetheless, we provide strong evidence that numerous proteins with functions in the cytosol are bound on the surface of *M. hyopneumoniae* where they are targets of endoproteolytic processing. We detected cleavage at S/T–X–F↓–X–D/E sites consistent with the hypothesis that the same enzyme that cleavages the P97 and P102 families is also targeting other surface accessible proteins.

Enrichment procedures such as TX-114 fractionation and affinity-capture chromatography techniques were useful for enriching the low-abundance proteome, delineating regions of proteins that bind host molecules and enriching for cleavage fragments, all of which provided clues to protein function. TX-114 extraction enriches for hydrophobic membrane proteins, which partition to the detergent phase [48]. As *M. hyopneumoniae* lacks a cell wall, the cell membrane is the mediator of contact between the bacteria and extracellular environment; hence, membrane-bound proteins are potentially valuable targets for vaccine and therapeutic development. While the TX-114 GeLC–MS/MS protocol detected the fewest protein identifications, at 206, it contributed five unique proteins to the overall analysis, all of which were uncharacterized proteins described as lipoproteins and/or predicted to contain transmembrane domains using TMpred. Overall, 26 of 50 *M. hyopneumoniae* lipoproteins were identified by all methods, and LC–MS/MS analysis of TX-114 solubilized proteins identified 22 of the 26.

While the precise functions of bacterial lipoproteins remain poorly understood, there is mounting evidence to suggest they are pathogen-associated molecular pattern (PAMP) molecules on the surface of Gram-positive bacteria. PAMPs are recognized by Toll-like receptors that trigger innate immune responses [49–52]. Most mycoplasma lipoproteins are surface-exposed with acyl groups anchoring these proteins in the cell membrane, where they are thought to function as cytadhesins, transport proteins or virulence factors with immunomodulatory capabilities [53]. P65 is an abundantly expressed, immunoreactive and lipolytic lipoprotein that selectively partitions to the detergent phase during extraction with TX-114 [38,54]. Schmidt *et al.* showed that anti-P65 antibodies inhibit the lipolytic activity of P65 and growth of *M. hyopneumoniae*, indicating that P65 performs a primary function on the external membrane surface by providing a source of essential lipids for growth [38]. It has also been suggested that P65 may alter surfactant properties in the lungs of pigs *in vivo* [38]. In our studies, P65 was recovered during affinity capture protocols using different host molecules as bait. Although these are preliminary data that require quantitative studies to confirm a direct role for P65 in these interactions, this suggests that P65 displays motifs that facilitate binding to a diverse range of host molecules. Consistent with these preliminary observations, we show here for the first time that P65 is a target of several processing events that generate cleavage fragments which are selectively retained during affinity chromatography using porcine epithelial cell surface proteins, fibronectin or porcine heparin as bait. The ability of the cleavage fragments of P65 to bind the same bait proteins as P65 lends weight to the hypothesis that the interactions with host molecules are direct and biologically relevant. Cleavage occurred at a number of sites in the P65 protein sequence, including at a phenylalanine residue within a S/T–X–F↓X–D/E motif; a known processing site in the P97 and P102 adhesin families [9–12,15,18,20–23]. An immunoblot of biotinylated cell surface *M. hyopneumoniae* strain J proteins fractionated using TX-114 that was probed with anti-P65 polyclonal antibodies identified a 65 kDa protein and numerous smaller mass fragments of P65 consistent with cleavage at several sites within the molecule. These data show that P65 and cleavage fragments of P65 reside on the surface of *M. hyopneumoniae*. Notably, there is clear evidence of a doublet at approximately 65 kDa (boxed in figure 2*b*) in the lane containing *M. hyopneumoniae* aqueous phase proteins. Previous studies have shown that P65 may undergo clipping at the N-terminus and be a target of further post-translational processing events [54]. Our data suggest that the doublet may represent forms of P65 that have lost the lipid anchor because they partitioned to the aqueous phase. If correct, these data suggest that a small lipopeptide similar to the macrophage-activating lipopeptide 2 (MALP-2) of *Mycoplasma fermentans* may be produced from P65.

Lipoproteins of mycoplasmal origin are known targets of post-translational processing events. The first 14 amino acids of MALP-404, a 41 kDa lipoprotein in *M. fermentans*, are removed by a post-translational cleavage event generating a 2 kDa MALP-2 lipopeptide. The C-terminal 39 kDa cleavage fragment (known as RF) that results from this cleavage event has been isolated from culture supernatants, but its function remains unknown [55]. Unlike RF, both MALP-2 and MALP-404 are lipid-modified and remain associated with the membrane of *M. fermentans*. MALP-2 is a potent immunomodulatory molecule that engages Toll-like receptor 2 [50]. Like the MALP-2 lipopeptide, the N-terminus of P65 may play a similar immunomodulatory role in *M. hyopneumoniae*; however, further studies are required to confirm this. Similarly, MGA0674 is an 82 kDa lipoprotein in *Mycoplasma gallisepticum* whose expression is elevated in virulent strain *R*_low_ compared with the attenuated vaccine strain F, suggesting that it may play a role in pathogenesis. MGA0674 is a target of a processing event at position 225 that releases a C-terminal 57 kDa fragment from the anchored N-terminal 22 kDa lipoprotein [56]. There are other reports of processing events that target lipoproteins in *Mycoplasma pneumoniae* but their functions have remained poorly characterized [57].

### 4.2. The extent of proteolytic processing in *Mycoplasma hyopneumoniae*

A significant number of the 35 proteins identified to be targets of post-translational processing were glycolytic enzymes and other metabolic proteins. Glycolytic enzymes are increasingly being identified as multitasking or moonlighting proteins in a wide range of organisms, including parasites [58], yeasts and fungi [59], mammalian cells [60], plants [61] and bacteria [62], and this is reflected in the range of entries seen in MultitaskProtDB [63]. In other members of the Mollicutes, proteins with canonical functions in the cytosol have also been found to be surface-exposed and interact with host components. For example, in *Mycoplasma pneumoniae*, elongation factor Tu (EfTu) and pyruvate dehydrogenase (PdhB) were identified as surface-exposed moonlighting proteins, through screening for fibronectin binding proteins by ligand blotting of whole cell lysates and fibronectin-coupled affinity chromatography, and their surface localization was confirmed by immunogold labelling and electron microscopy [64]. Further investigation of EfTu revealed specifically that the carboxyl-terminus was surface-exposed by immunogold labelling and responsible for fibronectin binding [65]. In *Mycoplasma genitalium*, glyceraldehyde-3-phosphate dehydrogenase was identified to be surface-exposed and bind mucin, probably functioning as an adhesin [66]. These proteins were identified here to be cleaved. Many processing events are likely to alter canonical (enzymatic) function and profoundly influence how cleavage fragments interact with the mycoplasma membrane and host molecules [67].

LDH is a highly immunogenic cytoplasmic protein involved in the glycolytic process of *M. hyopneumoniae* [68,69]. Here, we have identified LDH to be present at the cell surface both as a full-length molecule and as cleavage fragments. We identified a single putative cleavage site between amino acids 188–199 and the cleaved form of LDH is unlikely to carry out its primary function owing to significant structural alteration. In eukaryotic organisms, LDH has been recognized as a moonlighting protein, along with other glycolytic enzymes such as hexokinase, glyceraldehyde dehydrogenase and enolase, playing a role in transcriptional regulation [70]. LDH has also been identified as a single-stranded DNA-binding protein in eukaryotic cells [71,72]. In eukaryotic cells, this switch in function is likely to be due to translocation to the nucleus where these functions take place, possibly through post-translational modifications such as tyrosine phosphorylation [70,73,74]. It is possible that, as in eukaryotic cells, post-translational modifications may also affect localization and function of LDH in *M. hyopneumoniae*, or direct a subset towards processing. Intact LDH was identified from spots on two-dimensional gels between p*I* of 5.7 and 7.5. With a theoretical p*I* of 7.63, this indicates an acidic shift is likely to be caused by a variable degree of post-translational modification such as deamidation affecting a proportion of LDH [75]. LDH has also been identified from extracellular supernatants of various *Lactobacillus* and *Bifidobacterium* species from the honeybee *Apis mellifera* [76]. These lactic acid bacteria belong to the Firmicutes, and are genetically similar to the low G + C content mycoplasma species. It was hypothesized that LDH, once localized to the surface, could evolve alternative functions as a moonlighting protein, functioning as an auxiliary adhesin [76]. Indeed, we have also previously identified a glutamyl aminopeptidase from *M. hyopneumoniae*, MHJ_0125, which moonlights as a multifunctional adhesin at the cell surface [77], and a leucyl aminopeptidase, MHJ_0461, which functions as a multi-substrate peptidase and binds heparin, plasminogen and foreign DNA [47]. The cleavage fragments of LDH identified here bound to heparin, used as a structural mimic for glycosaminoglycans in the respiratory tract, and the N-terminal fragment also bound to fibronectin, an extracellular matrix component, indicating fragments may also have adhesin functions.

## 5. Conclusion

We identified 347 (52%) of the 672 putative ORFs predicted from the genome sequence of *M. hyopneumoniae* strain J. The proteome coverage from well-resolved two-dimensional gels, while low, is unsurprising. The limitations of two-dimensional gels are well documented, particularly considering the nature of sample preparation required, which limits the ability to retain and resolve very basic, acidic, small, large or hydrophobic proteins [43]. However, protein-centric, gel-based separations provide a technique complimentary to high-throughput two-dimensional LC–MS/MS protocols by maintaining mass and p*I* context, allowing the identification of cleavage products and the extent of proteolytic processing. We show for the first time that proteins with canonical functions in the cytosol that moonlight on the cell surface are also targets of endoproteolytic events. This describes a new dimension to protein moonlighting and suggests that much more biological information is inherent in proteins. While we cannot yet determine the exact nature of cleavage events as they occur *in vivo*, the analysis presented here is an important first step in determining physiologically relevant cleavage events. Cleavage events will undoubtedly complicate efforts to correlate the transcriptome with the proteome in future studies [78,79], and the protein-centric approaches presented here will provide a solid foundation for further investigation of post-translational processing in proteins involved in pathogenesis of *M. hyopneumoniae*, and will assist with delineating functionally important binding motifs.

## Supplementary Material

Table S1

## Supplementary Material

Figure S2

## Supplementary Material

Figure S3

## Supplementary Material

Figure S4

## References

[1] Razin S, Yogev D, Naot Y 1998 Molecular biology and pathogenicity of mycoplasmas. Microbiol. Mol. Biol. Rev. 62, 1094–1156. 9841667 10.1128/mmbr.62.4.1094-1156.1998 PMC98941

[2] Woese CR 1987 Bacterial evolution. Microbiol. Rev. 51, 221–271. 2439888 10.1128/mr.51.2.221-271.1987 PMC373105

[3] Minion FC, Lefkowitz EJ, Madsen ML, Cleary BJ, Swartzell SM, Mahairas GG 2004 The genome sequence of *Mycoplasma hyopneumoniae* strain 232, the agent of swine mycoplasmosis. J. Bacteriol. 186, 7123–7133. (doi:10.1128/jb.186.21.7123-7133.2004) 15489423 10.1128/JB.186.21.7123-7133.2004 PMC523201

[4] Clark LK, Armstrong CH, Freeman MJ, Scheidt AB, Sands-Freeman L, Knox K 1991 Investigating the transmission of *Mycoplasma hyopneumoniae* in a swine herd with enzootic pneumonia. Vet. Med. 86, 543–550.

[5] Liu W et al. 2011 Complete genome sequence of *Mycoplasma hyopneumoniae* strain 168. J. Bacteriol. 193, 1016–1017. (doi:10.1128/JB.01305-10) 21148737 10.1128/JB.01305-10 PMC3028675

[6] Vasconcelos AT et al. 2005 Swine and poultry pathogens: the complete genome sequences of two strains of *Mycoplasma hyopneumoniae* and a strain of *Mycoplasma synoviae*. J. Bacteriol. 187, 5568–5577. (doi:10.1128/JB.187.16.5568-5577.2005) 16077101 10.1128/JB.187.16.5568-5577.2005 PMC1196056

[7] Pendarvis K, Padula MP, Tacchi JL, Petersen AC, Djordjevic SP, Burgess SC, Minion FC 2014 Proteogenomic mapping of *Mycoplasma hyopneumoniae* virulent strain 232. BMC Genomics 15, 576 (doi:10.1186/1471-2164-15-576) 25005615 10.1186/1471-2164-15-576 PMC4102725

[8] Siqueira FM, Gerber AL, Guedes RL, Almeida LG, Schrank IS, Vasconcelos AT, Zaha A 2014 Unravelling the transcriptome profile of the swine respiratory tract mycoplasmas. PLoS ONE 9, e110327 (doi:10.1371/journal.pone.0110327) 25333523 10.1371/journal.pone.0110327 PMC4198240

[9] Bogema DR et al. 2012 Characterization of cleavage events in the multifunctional cilium adhesin Mhp684 (P146) reveals a mechanism by which *Mycoplasma hyopneumoniae* regulates surface topography. mBio 3 (doi:10.1128/mBio.00282-11) 10.1128/mBio.00282-11 PMC3322551 22493032

[10] Bogema DR et al. 2011 Sequence TTKF↓QE defines the site of proteolytic cleavage in Mhp683 protein, a novel glycosaminoglycan and cilium adhesin of *Mycoplasma hyopneumoniae*. J. Biol. Chem. 286, 41 217–41 229. (doi:10.1074/jbc.M111.226084) 10.1074/jbc.M111.226084 PMC3308835 21969369

[11] Deutscher AT, Jenkins C, Minion FC, Seymour LM, Padula MP, Dixon NE, Walker MJ, Djordjevic SP 2010 Repeat regions R1 and R2 in the P97 paralogue Mhp271 of *Mycoplasma hyopneumoniae* bind heparin, fibronectin and porcine cilia. Mol. Microbiol. 78, 444–458. (doi:10.1111/j.1365-2958.2010.07345.x) 20879998 10.1111/j.1365-2958.2010.07345.x

[12] Deutscher AT et al. 2012 Mycoplasma hyopneumoniae Surface proteins Mhp385 and Mhp384 bind host cilia and glycosaminoglycans and are endoproteolytically processed by proteases that recognize different cleavage motifs. J. Proteome Res. 11, 1924–1936. (doi:10.1021/pr201115v) 22229926 10.1021/pr201115v

[13] Hsu T, Artiushin S, Minion FC 1997 Cloning and functional analysis of the P97 swine cilium adhesin gene of *Mycoplasma hyopneumoniae*. J. Bacteriol. 179, 1317–1323. 9023217 10.1128/jb.179.4.1317-1323.1997 PMC178831

[14] Jenkins C, Wilton JL, Minion FC, Falconer L, Walker MJ, Djordjevic SP 2006 Two domains within the *Mycoplasma hyopneumoniae* cilium adhesin bind heparin. Infect. Immun. 74, 481–487. (doi:10.1128/iai.74.1.481-487.2006) 16369004 10.1128/IAI.74.1.481-487.2006 PMC1346629

[15] Seymour LM et al. 2010 A processed multidomain *Mycoplasma hyopneumoniae* adhesin binds fibronectin, plasminogen, and swine respiratory cilia. J. Biol. Chem. 285, 33 971–33 978. (doi:10.1074/jbc.M110.104463) 10.1074/jbc.M110.104463 PMC2962497 20813843

[16] Seymour LM, Falconer L, Deutscher AT, Minion FC, Padula MP, Dixon NE, Djordjevic SP, Walker MJ 2011 Mhp107 is a member of the multifunctional adhesin family of *Mycoplasma hyopneumoniae*. J. Biol. Chem. 286, 10 097–10 104. (doi:10.1074/jbc.M110.208140) 10.1074/jbc.M110.208140 PMC3060461 21245147

[17] Seymour LM et al. 2012 Mhp182 (P102) binds fibronectin and contributes to the recruitment of plasmin(ogen) to the *Mycoplasma hyopneumoniae* cell surface. Cell Microbiol. 14, 81–94. (doi:10.1111/j.1462-5822.2011.01702.x) 21951786 10.1111/j.1462-5822.2011.01702.x

[18] Wilton J et al. 2009 Mhp493 (P216) is a proteolytically processed, cilium and heparin binding protein of *Mycoplasma hyopneumoniae*. Mol. Microbiol. 71, 566–582. (doi:10.1111/j.1365-2958.2008.06546.x) 19040640 10.1111/j.1365-2958.2008.06546.x

[19] Zhang Q, Young TF, Ross RF 1994 Microtiter plate adherence assay and receptor analogs for *Mycoplasma hyopneumoniae*. Infect. Immun. 62, 1616–1622. 8168922 10.1128/iai.62.5.1616-1622.1994 PMC186367

[20] Raymond BB et al. 2014 Proteolytic processing of the cilium adhesin MHJ_0194 (P123) in *Mycoplasma hyopneumoniae* generates a functionally diverse array of cleavage fragments that bind multiple host molecules. Cell. Microbiol. 11, 1924–1936. (doi:10.1111/cmi.12377) 10.1111/cmi.12377 25293691

[21] Raymond BB, Tacchi JL, Jarocki VM, Minion FC, Padula MP, Djordjevic SP 2013 P159 from *Mycoplasma hyopneumoniae* binds porcine cilia and heparin and is cleaved in a manner akin to ectodomain shedding. J. Proteome Res. 12, 5891–5903. (doi:10.1021/pr400903s) 24195521 10.1021/pr400903s

[22] Tacchi JL, Raymond BB, Jarocki VM, Berry IJ, Padula MP, Djordjevic SP 2014 Cilium adhesin P216 (MHJ_0493) is a target of ectodomain shedding and aminopeptidase activity on the surface of *Mycoplasma hyopneumoniae*. J. Proteome Res. 13, 2920–2930. (doi:10.1021/pr500087c) 24804907 10.1021/pr500087c

[23] Djordjevic SP, Cordwell SJ, Djordjevic MA, Wilton J, Minion FC 2004 Proteolytic processing of the *Mycoplasma hyopneumoniae* cilium adhesin. Infect. Immun. 72, 2791–2802. (doi:10.1128/iai.72.5.2791-2802.2004) 15102789 10.1128/IAI.72.5.2791-2802.2004 PMC387856

[24] Moitinho-Silva L et al. 2013 *Mycoplasma hyopneumoniae* *in vitro* peptidase activities: identification and cleavage of kallikrein-kinin system-like substrates. Vet. Microbiol. 163, 264–273. (doi:10.1016/j.vetmic.2013.01.011) 23421966 10.1016/j.vetmic.2013.01.011

[25] Mann M, Kulak NA, Nagaraj N, Cox J 2013 The coming age of complete, accurate, and ubiquitous proteomes. Mol. Cell. 49, 583–590. (doi:10.1016/j.molcel.2013.01.029) 23438854 10.1016/j.molcel.2013.01.029

[26] Smith LM, Kelleher NL, Consortium for Top Down Proteomics. 2013 Proteoform: a single term describing protein complexity. Nat. Methods 10, 186–187. (doi:10.1038/nmeth.2369) 23443629 10.1038/nmeth.2369 PMC4114032

[27] Kelleher NL 2004 Top-down proteomics. Anal. Chem. 76, 197A–203A. (doi:10.1021/ac0415657) 15190879

[28] Friis NF 1975 Some recommendations concerning primary isolation of *Mycoplasma suipneumoniae* and *Mycoplasma flocculare* a survey. Nord Vet. Med. 27, 337–339. 1098011

[29] Scarman AL, Chin JC, Eamens GJ, Delaney SF, Djordjevic SP 1997 Identification of novel species-specific antigens of *Mycoplasma hyopneumoniae* by preparative SDS-PAGE ELISA profiling. Microbiology 143, 663–673. (doi:10.1099/00221287-143-2-663) 9043142 10.1099/00221287-143-2-663

[30] Jenkins C, Samudrala R, Geary SJ, Djordjevic SP 2008 Structural and functional characterization of an organic hydroperoxide resistance protein from *Mycoplasma gallisepticum*. J. Bacteriol. 190, 2206–2216. (doi:10.1128/JB.01685-07) 18192392 10.1128/JB.01685-07 PMC2258871

[31] Minion FC, Menon SA, Mahairas GG, Wannemuehler MJ 2003 Enhanced murine antigen-specific gamma interferon and immunoglobulin G2a responses by using mycobacterial ESAT-6 sequences in DNA vaccines. Infect. Immun. 71, 2239–2243. (doi:10.1128/IAI.71.4.2239-2243.2003) 12654848 10.1128/IAI.71.4.2239-2243.2003 PMC152058

[32] White MY, Brown DA, Sheng S, Cole RN, O'Rourke B, Van Eyk JE 2011 Parallel proteomics to improve coverage and confidence in the partially annotated *Oryctolagus cuniculus* mitochondrial proteome. Mol. Cell Proteomics 10, M110004291 (doi:10.1074/mcp.M110.004291) 10.1074/mcp.M110.004291 PMC3033681 21036924

[33] Wilkins MR, Gasteiger E, Bairoch A, Sanchez JC, Williams KL, Appel RD, Hochstrasser DF 1999 Protein identification and analysis tools in the ExPASy server. Methods Mol. Biol. 112, 531–552. 10027275 10.1385/1-59259-584-7:531

[34] Hofmann K, Stoffel W 1993 TMbase—a database of membrane spanning proteins segments. Biol. Chem. Hoppe-Seyler 374, 166.

[35] Obradovic Z, Peng K, Vucetic S, Radivojac P, Dunker AK 2005 Exploiting heterogeneous sequence properties improves prediction of protein disorder. Proteins 61(Suppl 7), 176–182. (doi:10.1002/prot.20735) 16187360 10.1002/prot.20735

[36] UniProt C 2015 UniProt: a hub for protein information. Nucleic Acids Res. 43, D204–D212. (doi:10.1093/nar/gku989) 25348405 10.1093/nar/gku989 PMC4384041

[37] Burnett TA et al. 2006 P159 is a proteolytically processed, surface adhesin of *Mycoplasma hyopneumoniae*: defined domains of P159 bind heparin and promote adherence to eukaryote cells. Mol. Microbiol. 60, 669–686. (doi:10.1111/j.1365-2958.2006.05139.x) 16629669 10.1111/j.1365-2958.2006.05139.x

[38] Schmidt JA, Browning GF, Markham PF 2004 Mycoplasma hyopneumoniae p65 surface lipoprotein is a lipolytic enzyme with a preference for shorter-chain fatty acids. J. Bacteriol. 186, 5790–5798. (doi:10.1128/JB.186.17.5790-5798.2004) 15317784 10.1128/JB.186.17.5790-5798.2004 PMC516823

[39] Ferron F, Longhi S, Canard B, Karlin D 2006 A practical overview of protein disorder prediction methods. Proteins 65, 1–14. (doi:10.1002/prot.21075) 16856179 10.1002/prot.21075

[40] Tacchi JL, Raymond BBA, Djordjevic SP Characterisation of proteins on the surface of *Mycoplasma hyopneumoniae*. In preparation.

[41] Lopez JL 2007 Two-dimensional electrophoresis in proteome expression analysis. J. Chromatogr. B 849, 190–202. (doi:10.1016/j.jchromb.2006.11.049) 10.1016/j.jchromb.2006.11.049 17188947

[42] Ly L, Wasinger VC 2011 Protein and peptide fractionation, enrichment and depletion: tools for the complex proteome. Proteomics 11, 513–534. (doi:10.1002/pmic.201000394) 21241016 10.1002/pmic.201000394

[43] Maillet I, Berndt P, Malo C, Rodriguez S, Brunisholz RA, Pragai Z, Arnold S, Langen H, Wyss M 2007 From the genome sequence to the proteome and back: evaluation of *E. coli* genome annotation with a 2-D gel-based proteomics approach. Proteomics 7, 1097–1106. (doi:10.1002/pmic.200600599) 17366475 10.1002/pmic.200600599

[44] Reinders J, Zahedi RP, Pfanner N, Meisinger C, Sickmann A 2006 Toward the complete yeast mitochondrial proteome: multidimensional separation techniques for mitochondrial proteomics. J. Proteome Res. 5, 1543–1554. (doi:10.1021/pr050477f) 16823961 10.1021/pr050477f

[45] Bendtsen JD, Kiemer L, Fausboll A, Brunak S 2005 Non-classical protein secretion in bacteria. BMC Microbiol. 5, 58 (doi:10.1186/1471-2180-5-58) 16212653 10.1186/1471-2180-5-58 PMC1266369

[46] Henderson B, Martin A 2011 Bacterial virulence in the moonlight: multitasking bacterial moonlighting proteins are virulence determinants in infectious disease. Infect. Immun. 79, 3476–3491. (doi:10.1128/IAI.00179-11) 21646455 10.1128/IAI.00179-11 PMC3165470

[47] Jarocki VM, Santos J, Tacchi JL, Raymond BBA, Deutscher AT, Jenkins C, Padula MP, Djordjevic SP 2015 MHJ_0461 is a multifunctional leucine aminopeptidase on the surface of *Mycoplasma hyopneumoniae*. Open Biol. 5, 140175 (doi:10.1098/rsob.140175) 25589579 10.1098/rsob.140175 PMC4313372

[48] Wise KS, Kim MF 1987 Major membrane surface proteins of *Mycoplasma hyopneumoniae* selectively modified by covalently bound lipid. J. Bacteriol. 169, 5546–5555. 3680170 10.1128/jb.169.12.5546-5555.1987 PMC213984

[49] Hashimoto M, Tawaratsumida K, Kariya H, Aoyama K, Tamura T, Suda Y 2006 Lipoprotein is a predominant Toll-like receptor 2 ligand in *Staphylococcus aureus* cell wall components. Int. Immunol. 18, 355–362. (doi:10.1093/intimm/dxh374) 16373361 10.1093/intimm/dxh374

[50] Muhlradt PF, Kiess M, Meyer H, Sussmuth R, Jung G 1997 Isolation, structure elucidation, and synthesis of a macrophage stimulatory lipopeptide from *Mycoplasma fermentans* acting at picomolar concentration. J. Exp. Med. 185, 1951–1958. (doi:10.1084/jem.185.11.1951) 9166424 10.1084/jem.185.11.1951 PMC2196331

[51] Ozinsky A, Underhill DM, Fontenot JD, Hajjar AM, Smith KD, Wilson CB, Schroeder L, Aderem A 2000 The repertoire for pattern recognition of pathogens by the innate immune system is defined by cooperation between toll-like receptors. Proc. Natl Acad. Sci. USA 97, 13 766–13 771. (doi:10.1073/pnas.250476497) 10.1073/pnas.250476497 PMC17650 11095740

[52] Takeuchi O, Kawai T, Muhlradt PF, Morr M, Radolf JD, Zychlinsky A, Takeda K, Akira S 2001 Discrimination of bacterial lipoproteins by Toll-like receptor 6. Int. Immunol. 13, 933–940. (doi:10.1093/intimm/13.7.933) 11431423 10.1093/intimm/13.7.933

[53] Browning GF, Marenda MS, Noormohammadi AH, Markham PF 2011 The central role of lipoproteins in the pathogenesis of mycoplasmoses. Vet. Microbiol. 153, 44–50. (doi:10.1016/j.vetmic.2011.05.031) 21684094 10.1016/j.vetmic.2011.05.031

[54] Kim MF, Heidari MB, Stull SJ, McIntosh MA, Wise KS 1990 Identification and mapping of an immunogenic region of *Mycoplasma hyopneumoniae* p65 surface lipoprotein expressed in *Escherichia coli* from a cloned genomic fragment. Infect. Immun. 58, 2637–2643. 1695206 10.1128/iai.58.8.2637-2643.1990 PMC258866

[55] Davis KL, Wise KS 2002 Site-specific proteolysis of the MALP-404 lipoprotein determines the release of a soluble selective lipoprotein-associated motif-containing fragment and alteration of the surface phenotype of *Mycoplasma fermentans*. Infect. Immun. 70, 1129–1135. (doi:10.1128/IAI.70.3.1129-1135.2002) 11854192 10.1128/IAI.70.3.1129-1135.2002 PMC127791

[56] Szczepanek SM, Frasca S Jr, Schumacher VL, Liao X, Padula M, Djordjevic SP, Geary SJ 2010 Identification of lipoprotein MslA as a neoteric virulence factor of *Mycoplasma gallisepticum*. Infect. Immun. 78, 3475–3483. (doi:10.1128/IAI.00154-10) 20515935 10.1128/IAI.00154-10 PMC2916287

[57] Regula JT, Ueberle B, Boguth G, Gorg A, Schnolzer M, Herrmann R, Frank R 2000 Towards a two-dimensional proteome map of *Mycoplasma pneumoniae*. Electrophoresis 21, 3765–3780. (doi:10.1002/1522-2683(200011)21:17<3765::AID-ELPS3765>3.0.CO;2-6) 11271496 10.1002/1522-2683(200011)21:17<3765::AID-ELPS3765>3.0.CO;2-6

[58] Gomez-Arreaza A, Acosta H, Quinones W, Concepcion JL, Michels PAM, Avilan L 2014 Extracellular functions of glycolytic enzymes of parasites: Unpredicted use of ancient proteins. Mol. Biochem. Parasitol. 193, 75–81. (doi:10.1016/j.molbiopara.2014.02.005) 24602601 10.1016/j.molbiopara.2014.02.005

[59] Ikeda R, Ichikawa T 2014 Interaction of surface molecules on *Cryptococcus neoformans* with plasminogen. FEMS Yeast Res. 14, 445–450. (doi:10.1111/1567-1364.12131) 24373348 10.1111/1567-1364.12131 PMC4282087

[60] Petit FM, Serres C, Bourgeon F, Pineau C, Auer J 2013 Identification of sperm head proteins involved in zona pellucida binding. Hum. Reprod. 28, 852–865. (doi:10.1093/humrep/des452) 23355646 10.1093/humrep/des452

[61] Zaffagnini M, Fermani S, Costa A, Lemaire SD, Trost P 2013 Plant cytoplasmic GAPDH: redox post-translational modifications and moonlighting properties. Front. Plant Sci. 4, 450 (doi:10.3389/Fpls.2013.00450) 24282406 10.3389/fpls.2013.00450 PMC3824636

[62] Wang GQ, Xia Y, Cui J, Gu ZN, Song YD, Chen YQ, Chen HQ, Zhang H, Chen W 2014 The roles of moonlighting proteins in bacteria. Curr. Issues Mol. Biol. 16, 15–22. 23872606

[63] Hernandez S, Ferragut G, Amela I, Perez-Pons J, Pinol J, Mozo-Villarias A, Cedano J, Querol E 2014 MultitaskProtDB: a database of multitasking proteins. Nucleic Acids Res. 42, D517–D520. (doi:10.1093/Nar/Gkt1153) 24253302 10.1093/nar/gkt1153 PMC3965044

[64] Dallo SF, Kannan TR, Blaylock MW, Baseman JB 2002 Elongation factor Tu and E1 beta subunit of pyruvate dehydrogenase complex act as fibronectin binding proteins in *Mycoplasma pneumoniae*. Mol. Microbiol. 46, 1041–1051. (doi:10.1046/j.1365-2958.2002.03207.x) 12421310 10.1046/j.1365-2958.2002.03207.x

[65] Balasubramanian S, Kannan TR, Baseman JB 2008 The surface-exposed carboxyl region of *Mycoplasma pneumoniae* elongation factor Tu interacts with fibronectin. Infect. Immun. 76, 3116–3123. (doi:10.1128/IAI.00173-08) 18411296 10.1128/IAI.00173-08 PMC2446705

[66] Alvarez RA, Blaylock MW, Baseman JB 2003 Surface localized glyceraldehyde-3-phosphate dehydrogenase of *Mycoplasma genitalium* binds mucin. Mol. Microbiol. 48, 1417–1425. (doi:10.1046/j.1365-2958.2003.03518.x) 12787366 10.1046/j.1365-2958.2003.03518.x

[67] Sueyoshi N, Nimura T, Onouchi T, Baba H, Takenaka S, Ishida A, Kameshita I 2012 Functional processing of nuclear Ca^2+^/calmodulin-dependent protein kinase phosphatase (CaMKP-N): evidence for a critical role of proteolytic processing in the regulation of its catalytic activity, subcellular localization and substrate targeting *in vivo*. Arch. Biochem. Biophys. 517, 43–52. (doi:10.1016/j.abb.2011.10.017) 22100705 10.1016/j.abb.2011.10.017

[68] Frey J, Haldimann A, Kobisch M, Nicolet J 1994 Immune response against the l-lactate dehydrogenase of *Mycoplasma hyopneumoniae* in enzootic pneumonia of swine. Microb. Pathog. 17, 313–322. (doi:10.1006/mpat.1994.1077) 7723658 10.1006/mpat.1994.1077

[69] Haldimann A, Nicolet J, Frey J 1993 DNA sequence determination and biochemical analysis of the immunogenic protein P36, the lactate dehydrogenase (LDH) of *Mycoplasma hyopneumoniae*. J. Gen. Microbiol. 139, 317–323. (doi:10.1099/00221287-139-2-317) 7679720 10.1099/00221287-139-2-317

[70] Kim JW, Dang CV 2005 Multifaceted roles of glycolytic enzymes. Trends Biochem. Sci. 30, 142–150. (doi:10.1016/j.tibs.2005.01.005) 15752986 10.1016/j.tibs.2005.01.005

[71] Cattaneo A, Biocca S, Corvaja N, Calissano P 1985 Nuclear localization of a lactic dehydrogenase with single-stranded DNA-binding properties. Exp. Cell Res. 161, 130–140. (doi:10.1016/0014-4827(85)90497-5) 3902489 10.1016/0014-4827(85)90497-5

[72] Grosse F, Nasheuer HP, Scholtissek S, Schomburg U 1986 Lactate dehydrogenase and glyceraldehyde-phosphate dehydrogenase are single-stranded DNA-binding proteins that affect the DNA-polymerase-alpha-primase complex. Eur. J. Biochem. 160, 459–467. (doi:10.1111/j.1432-1033.1986.tb10062.x) 3536507 10.1111/j.1432-1033.1986.tb10062.x

[73] Zhong XH, Howard BD 1990 Phosphotyrosine-containing lactate dehydrogenase is restricted to the nuclei of PC12 pheochromocytoma cells. Mol. Cell Biol. 10, 770–776. (doi:10.1128/MCB.10.2.770) 1689001 10.1128/mcb.10.2.770-776.1990 PMC360877

[74] Cooper JA, Esch FS, Taylor SS, Hunter T 1984 Phosphorylation sites in enolase and lactate dehydrogenase utilized by tyrosine protein kinases *in vivo* and *in vitro*. J. Biol. Chem. 259, 7835–7841. 6330085

[75] Sarioglu H, Lottspeich F, Walk T, Jung G, Eckerskorn C 2000 Deamidation as a widespread phenomenon in two-dimensional polyacrylamide gel electrophoresis of human blood plasma proteins. Electrophoresis 21, 2209–2218. (doi:10.1002/1522-2683(20000601)21:11<2209::AID-ELPS2209>3.0.CO;2-T) 10892731 10.1002/1522-2683(20000601)21:11<2209::AID-ELPS2209>3.0.CO;2-T

[76] Butler E, Alsterfjord M, Olofsson TC, Karlsson C, Malmstrom J, Vasquez A 2013 Proteins of novel lactic acid bacteria from *Apis mellifera* mellifera: an insight into the production of known extra-cellular proteins during microbial stress. BMC Microbiol. 13, 235 (doi:10.1186/1471-2180-13-235) 24148670 10.1186/1471-2180-13-235 PMC4015849

[77] Robinson MW et al. 2013 MHJ_0125 is an M42 glutamyl aminopeptidase that moonlights as a multifunctional adhesin on the surface of *Mycoplasma hyopneumoniae*. Open Biol. 3, 130017 (doi:10.1098/rsob.130017) 23594879 10.1098/rsob.130017 PMC3718333

[78] Gunawardana Y, Niranjan M 2013 Bridging the gap between transcriptome and proteome measurements identifies post-translationally regulated genes. Bioinformatics 29, 3060–3066. (doi:10.1093/bioinformatics/btt537) 24045772 10.1093/bioinformatics/btt537

[79] Olivares-Hernandez R, Bordel S, Nielsen J 2011 Codon usage variability determines the correlation between proteome and transcriptome fold changes. BMC Syst. Biol. 5, 33 (doi:10.1186/1752-0509-5-33) 21352515 10.1186/1752-0509-5-33 PMC3058016

