# Supplementary material for: Post-translational processing targets functionally diverse proteins in Mycoplasma hyopneumoniae
Source: Open Biol. 2016 Feb 10;6(2):150210. doi: 10.1098/rsob.150210 (PMC4772806; doi:10.1098/rsob.150210)
Supplement: Figure S4 [file rsob150210supp4.pdf]

Supplementary Figure S4:

Spectra and fragmentation tables identifying single-peptide protein identifications

ID #	Accession	m/z	z	Error (ppm)	Peptide	Score
285	Q4A9X7	985.07	2	74	QLVELYDFEISELENK	60
335	Q4AA71	781.48	2	63	IWLISEGIENLFK	45.7
339	Q4AAC5	747.87	2	4.9	VDDENLDSIFSK	70.2
344	Q4AA34	630.37	2	2.1	VVGISSLTTLNR	51.8
345	A4Q7T6	985.07	2	74	QLVELYDFESELENK	68.6
347	Q4A9M3	822.94	2	10	YNLSNFFILISDAK	51.4

#285 Q4A9X7 MHJ_0356 Putative P37-like ABC transporter substrate-binding lipoprotein – identified from TX-114 1DGE LC-MS/MS (Slice 14)

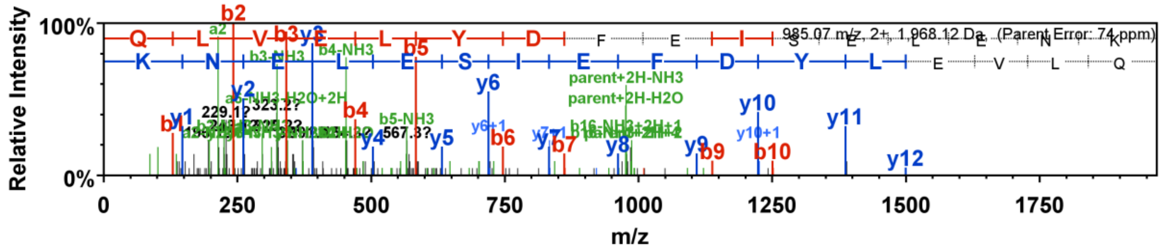

B	B Ions	B+2H	B-NH3	B-H2O	AA	Y Ions	Y+2H	Y-NH3	Y-H2O	Y
1	129.1		112.0		Q	1,969.0	985.0	1,952.0	1,951.0	16
2	242.1		225.1		L	1,840.9	921.0	1,823.9	1,822.9	15
3	341.2		324.2		V	1,727.8	864.4	1,710.8	1,709.8	14
4	470.3		453.2	452.3	E	1,628.8	814.9	1,611.7	1,610.8	13
5	583.3		566.3	565.3	L	1,499.7	750.4	1,482.7	1,481.7	12
6	746.4	373.7	729.4	728.4	Y	1,386.6	693.8	1,369.6	1,368.6	11
7	861.4	431.2	844.4	843.4	D	1,223.6	612.3	1,206.6	1,205.6	10
8	1,008.5	504.8	991.5	990.5	F	1,108.6	554.8	1,091.5	1,090.5	9
9	1,137.5	569.3	1,120.5	1,119.5	E	961.5	481.2	944.5	943.5	8
10	1,250.6	625.8	1,233.6	1,232.6	I	832.4	416.7	815.4	814.4	7
11	1,337.7	669.3	1,320.6	1,319.7	S	719.4	360.2	702.3	701.3	6
12	1,466.7	733.9	1,449.7	1,448.7	E	632.3		615.3	614.3	5
13	1,579.8	790.4	1,562.8	1,561.8	L	503.3		486.3	485.3	4
14	1,708.8	854.9	1,691.8	1,690.8	E	390.2		373.2	372.2	3
15	1,822.9	911.9	1,805.8	1,804.9	N	261.2		244.1		2
16	1,969.0	985.0	1,952.0	1,951.0	K	147.1		130.1		1

#335 Q4AA71 MHJ_0259 UPF0348 protein – identified from TX-114 1DGE LC-MS/MS (Slice 10)

Correct mass context for predicted intact protein

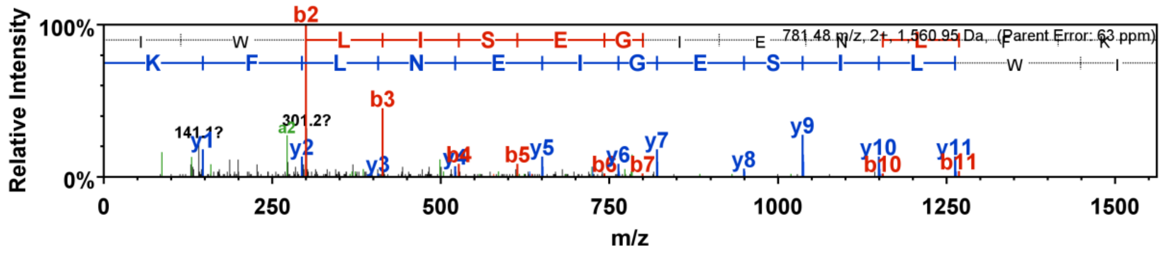

B	B Ions	B+2H	B-NH3	B-H2O	AA	Y Ions	Y+2H	Y-NH3	Y-H2O	Y
1	114.1				I	1,561.9	781.4	1,544.8	1,543.9	13
2	300.2				W	1,448.8	724.9	1,431.8	1,430.8	12
3	413.3				L	1,262.7	631.9	1,245.7	1,244.7	11
4	526.3				I	1,149.6	575.3	1,132.6	1,131.6	10
5	613.4			595.4	S	1,036.5	518.8	1,019.5	1,018.5	9
6	742.4	371.7		724.4	E	949.5	475.3	932.5	931.5	8
7	799.4	400.2		781.4	G	820.5	410.7	803.4	802.4	7
8	912.5	456.8		894.5	I	763.4	382.2	746.4	745.4	6
9	1,041.6	521.3		1,023.6	E	650.4		633.3	632.3	5
10	1,155.6	578.3	1,138.6	1,137.6	N	521.3		504.3		4
11	1,268.7	634.8	1,251.7	1,250.7	L	407.3		390.2		3
12	1,415.8	708.4	1,398.7	1,397.7	F	294.2		277.2		2
13	1,561.9	781.4	1,544.8	1,543.9	K	147.1		130.1		1

#339 Q4AAC5 MHJ_0205 tRNA modification GTPase MnmE (EC 3.6.-.-) – identified from QTOF 1DGE LC-MS/MS (Slice 7)

Correct mass context for predicted intact protein

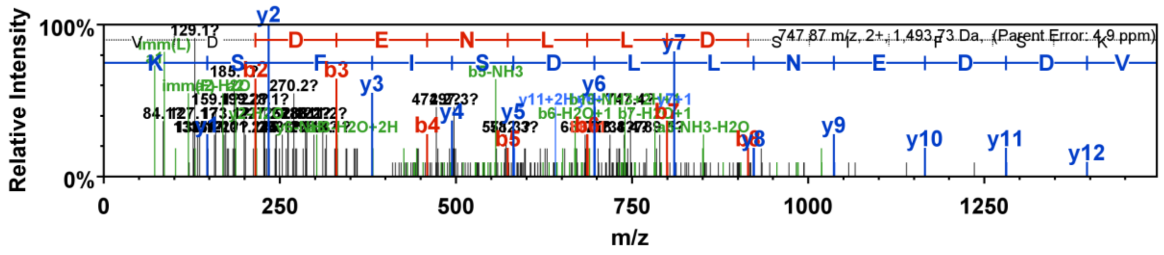

B	B Ions	B+2H	B-NH3	B-H2O	AA	Y Ions	Y+2H	Y-NH3	Y-H2O	Y
1	100.1				V	1,494.7	747.9	1,477.7	1,476.7	13
2	215.1			197.1	D	1,395.7	698.3	1,378.6	1,377.7	12
3	330.1			312.1	D	1,280.6	640.8	1,263.6	1,262.6	11
4	459.2			441.2	E	1,165.6	583.3	1,148.6	1,147.6	10
5	573.2		556.2	555.2	N	1,036.6	518.8	1,019.5	1,018.6	9
6	686.3	343.7	669.3	668.3	L	922.5	461.8	905.5	904.5	8
7	799.4	400.2	782.4	781.4	L	809.4	405.2	792.4	791.4	7
8	914.4	457.7	897.4	896.4	D	696.4	348.7	679.3	678.3	6
9	1,001.4	501.2	984.4	983.4	S	581.3		564.3	563.3	5
10	1,114.5	557.8	1,097.5	1,096.5	I	494.3		477.3	476.3	4
11	1,261.6	631.3	1,244.6	1,243.6	F	381.2		364.2	363.2	3
12	1,348.6	674.8	1,331.6	1,330.6	S	234.1		217.1	216.1	2
13	1,494.7	747.9	1,477.7	1,476.7	K	147.1		130.1		1

#344 Q4AA34 MHJ_0297 Putative ABC transporter ATP-binding protein – identified from QTOF overload lane (Slice 1)

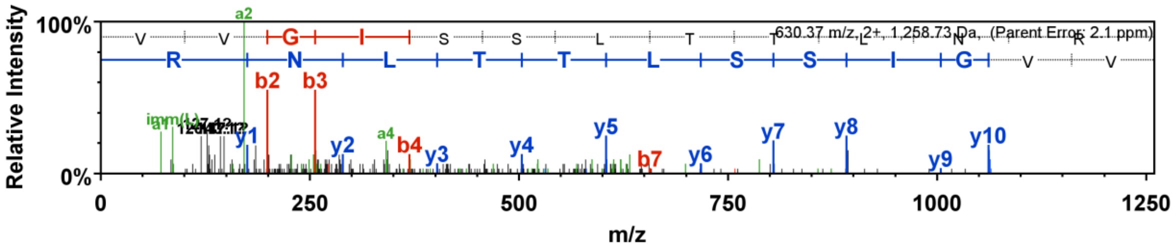

B	B Ions	B+2H	B-NH3	B-H2O	AA	Y Ions	Y+2H	Y-NH3	Y-H2O	Y
1	100.1				V	1,259.7	630.4	1,242.7	1,241.7	12
2	199.1				V	1,160.7	580.8	1,143.6	1,142.7	11
3	256.2				G	1,061.6	531.3	1,044.6	1,043.6	10
4	369.2				I	1,004.6	502.8	987.5	986.6	9
5	456.3			438.3	S	891.5	446.2	874.5	873.5	8
6	543.3	272.2		525.3	S	804.5	402.7	787.4	786.4	7
7	656.4	328.7		638.4	L	717.4	359.2	700.4	699.4	6
8	757.4	379.2		739.4	T	604.3		587.3	586.3	5
9	858.5	429.8		840.5	T	503.3		486.3	485.3	4
10	971.6	486.3		953.6	L	402.2		385.2		3
11	1,085.6	543.3	1,068.6	1,067.6	N	289.2		272.1		2
12	1,259.7	630.4	1,242.7	1,241.7	R	175.1		158.1		1

#345 A4Q7T6 MHJ_0696 Uncharacterised protein – identified from TX-114 1DGE LC-MS/MS (Slice 14). Correct mass context for predicted intact protein

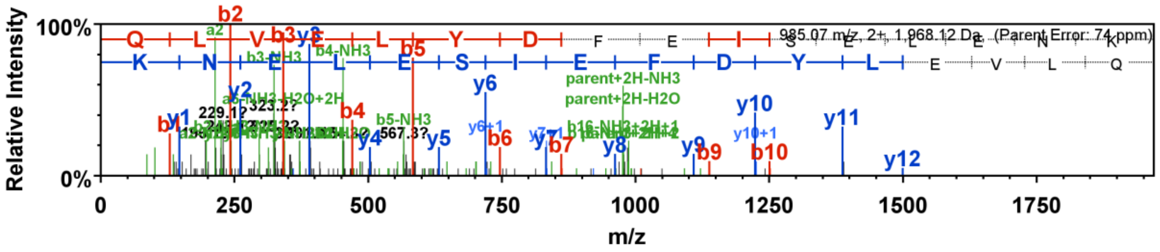

B	B Ions	B+2H	B-NH3	B-H2O	AA	Y Ions	Y+2H	Y-NH3	Y-H2O	Y
1	129.1		112.0		Q	1,969.0	985.0	1,952.0	1,951.0	16
2	242.1		225.1		L	1,840.9	921.0	1,823.9	1,822.9	15
3	341.2		324.2		V	1,727.8	864.4	1,710.8	1,709.8	14
4	470.3		453.2	452.3	E	1,628.8	814.9	1,611.7	1,610.8	13
5	583.3		566.3	565.3	L	1,499.7	750.4	1,482.7	1,481.7	12
6	746.4	373.7	729.4	728.4	Y	1,386.6	693.8	1,369.6	1,368.6	11
7	861.4	431.2	844.4	843.4	D	1,223.6	612.3	1,206.6	1,205.6	10
8	1,008.5	504.8	991.5	990.5	F	1,108.6	554.8	1,091.5	1,090.5	9
9	1,137.5	569.3	1,120.5	1,119.5	E	961.5	481.2	944.5	943.5	8
10	1,250.6	625.8	1,233.6	1,232.6	I	832.4	416.7	815.4	814.4	7
11	1,337.7	669.3	1,320.6	1,319.7	S	719.4	360.2	702.3	701.3	6
12	1,466.7	733.9	1,449.7	1,448.7	E	632.3		615.3	614.3	5
13	1,579.8	790.4	1,562.8	1,561.8	L	503.3		486.3	485.3	4
14	1,708.8	854.9	1,691.8	1,690.8	E	390.2		373.2	372.2	3
15	1,822.9	911.9	1,805.8	1,804.9	N	261.2		244.1		2
16	1,969.0	985.0	1,952.0	1,951.0	K	147.1		130.1		1

#347 Q4A9M3 MHJ_0462 tRNA (guanine-N(7)-)-methyltransferase (EC 2.1.1.33) (tRNA (guanine(46)-N(7))-methyltransferase) – identified from QTOF overload lane (Slice 25)

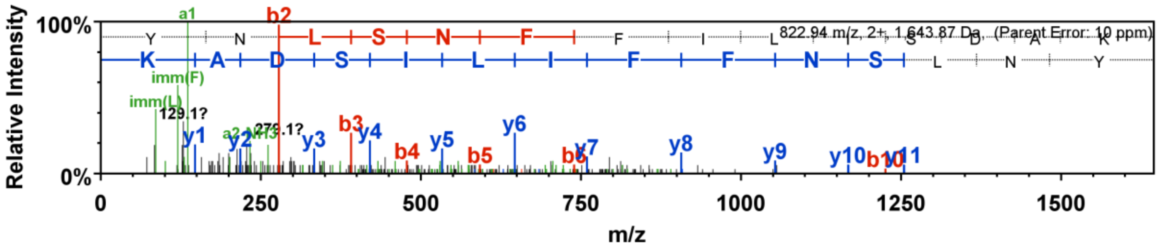

B	B Ions	B+2H	B-NH3	B-H2O	AA	Y Ions	Y+2H	Y-NH3	Y-H2O	Y
1	164.1				Y	1,644.9	822.9	1,627.8	1,626.9	14
2	278.1		261.1		N	1,481.8	741.4	1,464.8	1,463.8	13
3	391.2		374.2		L	1,367.8	684.4	1,350.7	1,349.7	12
4	478.2		461.2	460.2	S	1,254.7	627.8	1,237.6	1,236.7	11
5	592.3		575.2	574.3	N	1,167.6	584.3	1,150.6	1,149.6	10
6	739.3	370.2	722.3	721.3	F	1,053.6	527.3	1,036.6	1,035.6	9
7	886.4	443.7	869.4	868.4	F	906.5	453.8	889.5	888.5	8
8	999.5	500.3	982.5	981.5	I	759.5	380.2	742.4	741.5	7
9	1,112.6	556.8	1,095.6	1,094.6	L	646.4	323.7	629.4	628.4	6
10	1,225.7	613.3	1,208.6	1,207.7	I	533.3		516.3	515.3	5
11	1,312.7	656.9	1,295.7	1,294.7	S	420.2		403.2	402.2	4
12	1,427.7	714.4	1,410.7	1,409.7	D	333.2		316.2	315.2	3
13	1,498.8	749.9	1,481.7	1,480.7	A	218.1		201.1		2
14	1,644.9	822.9	1,627.8	1,626.9	K	147.1		130.1		1

Spectra and fragmentation tables identifying semi-tryptic peptide identifications mapping to P65 (MHJ_0656, Q4A932)

Site	Peptide sequence	Score	E value	Identified	No.
1-1	K.^{90}NSLVSYDNLAISGTTTE^{106}.N	61	0.0011	Peptide-centric*	5
1-2	E.^{107}NWLYLLNPTK^{116}.Y	50	0.011	Peptide-centric	1
1-3	E.^{107}NWLYLLNPTKYPNGK^{121}.M + Deamidated (N)	51	0.011	Peptide-centric	1
2-1	M.^{168}SVGANDPFLAIFNEFK^{184}.K	74	4.4e-06	WCL Ion Trap	2
2-2	M.^{168}SVGANDPFLAIFNEFK^{184}.K	50	0.0025	TX114 Q-TOF	1
2-3	M.^{168}SVGANDPFLAIFNEFK^{184}.K	82	0.011	Gel Spots	1
4-1	F.^{504}FAELNTDQEIK^{514}.E	56	0.0024	WCL Q-TOF	1
4-2	F.^{504}FAELNTDQEIK^{514}.E	90	0.0014	Gel Spots	6
4-3	F.^{504}FAELNTDQEIK^{514}.E + Deamidated (Q)	108	2.8e-05	Gel Spots	2

Table shows site of cleavage that semi-tryptic peptide denotes in P65 according to figure 2, with peptide sequence showing amino acid positions and semi-tryptic terminus (underlined). No. indicates the number of times a peptide was identified by a given method. In the case where peptides were identified multiple times or in multiple runs, only the highest-scoring peptide is shown. *Peptide centric methods described below.

#1-1. Peptide-centric analysis
MS/MS Fragmentation of **(K)^{90}NSLVSYDNLAISGTTTE^{106}(N)**
Mascot Ion Score: 61 E-value: 0.0011

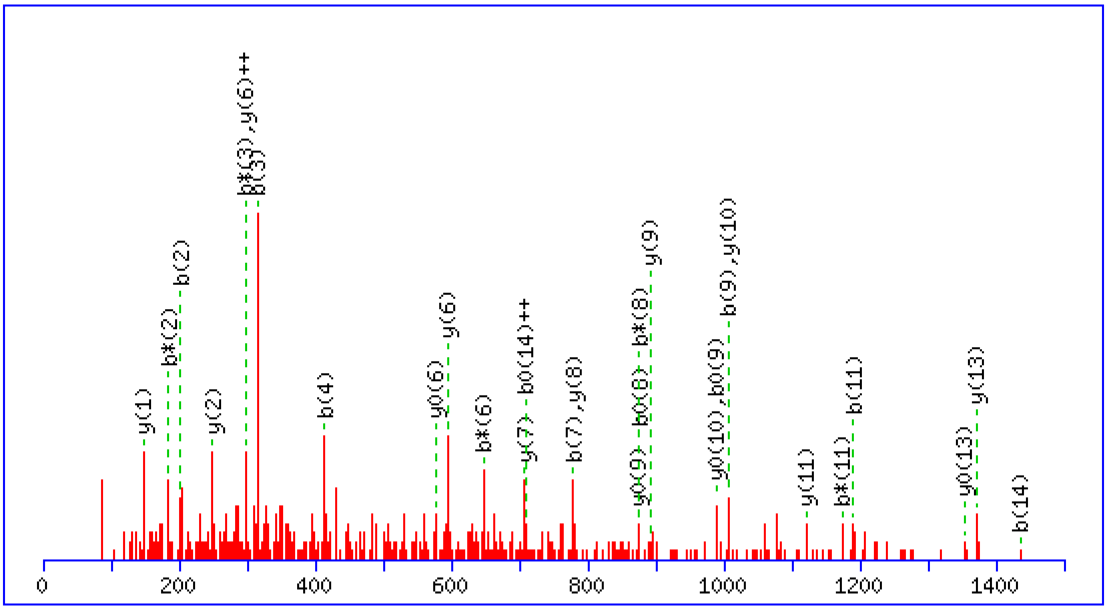

#	b	b^{++}	b*	b^{*++}	b^{0}	b^{0++}	Seq.	y	y^{++}	y*	y^{*++}	y^{0}	y^{0++}	#
1	115.0502	58.0287	98.0237	49.5155			N							17
2	***202.0822***	101.5448	185.0557	93.0315	184.0717	92.5395	S	1670.8119	835.9096	1653.7854	827.3963	1652.8014	826.9043	16
3	***315.1663***	158.0868	298.1397	149.5735	297.1557	149.0815	L	1583.7799	792.3936	1566.7534	783.8803	1565.7693	783.3883	15
4	***414.2347***	207.6210	397.2082	199.1077	396.2241	198.6157	V	1470.6958	735.8516	1453.6693	727.3383	1452.6853	726.8463	14
5	501.2667	251.1370	484.2402	242.6237	483.2562	242.1317	S	***1371.6274***	686.3174	1354.6009	677.8041	1353.6169	677.3121	13
6	664.3301	332.6687	647.3035	324.1554	646.3195	323.6634	Y	1284.5954	642.8013	1267.5689	634.2881	1266.5848	633.7961	12
7	***779.3570***	390.1821	762.3305	381.6689	761.3464	381.1769	D	***1121.5321***	561.2697	1104.5055	552.7564	1103.5215	552.2644	11

8	893.3999	447.2036	876.3734	438.6903	875.3894	438.1983	N	1006.5051	503.7562	989.4786	495.2429	988.4946	494.7509	10
9	1006.4840	503.7456	989.4575	495.2324	988.4734	494.7404	L	892.4622	446.7347			874.4516	437.7295	9
10	1077.5211	539.2642	1060.4946	530.7509	1059.5105	530.2589	A	779.3781	390.1927			761.3676	381.1874	8
11	1190.6052	595.8062	1173.5786	587.2930	1172.5946	586.8009	I	708.3410	354.6742			690.3305	345.6689	7
12	1277.6372	639.3222	1260.6107	630.8090	1259.6266	630.3170	S	595.2570	298.1321			577.2464	289.1268	6
13	1334.6587	667.8330	1317.6321	659.3197	1316.6481	658.8277	G	508.2249	254.6161			490.2144	245.6108	5
14	1435.7063	718.3568	1418.6798	709.8435	1417.6958	709.3515	T	451.2035	226.1054			433.1929	217.1001	4
15	1536.7540	768.8807	1519.7275	760.3674	1518.7435	759.8754	T	350.1558	175.5815			332.1452	166.5763	3
16	1637.8017	819.4045	1620.7752	810.8912	1619.7911	810.3992	T	249.1081	125.0577			231.0975	116.0524	2
17							E	148.0604	74.5339			130.0499	65.5286	1

#1-2. Peptide-centric analysis
MS/MS Fragmentation of **E.^{107}NWLYLLNPTK^{116}.Y**
Mascot Ion Score: 50 E-value: 0.011

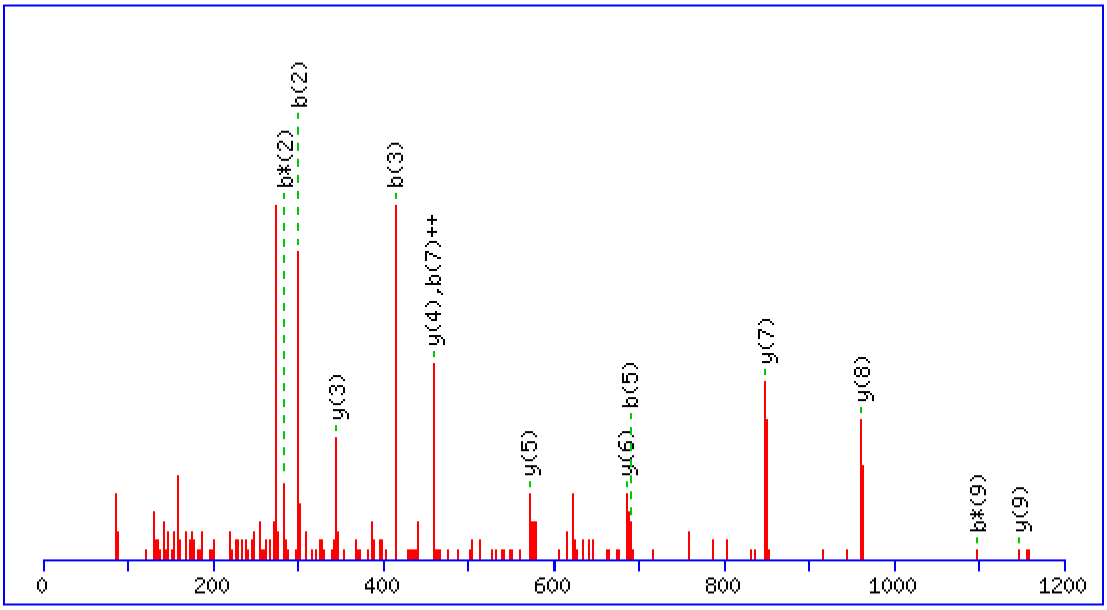

#	b	b++	b*	b*++	b0	b0++	Seq.	y	y++	y*	y*++	y0	y0++	#
1	115.0502	58.0287	98.0237	49.5155			N							10
2	301.1295	151.0684	284.1030	142.5551			W	1147.6510	574.3291	1130.6245	565.8159	1129.6404	565.3239	9
3	414.2136	207.6104	397.1870	199.0972			L	961.5717	481.2895	944.5451	472.7762	943.5611	472.2842	8
4	577.2769	289.1421	560.2504	280.6288			Y	848.4876	424.7475	831.4611	416.2342	830.4771	415.7422	7
5	690.3610	345.6841	673.3344	337.1709			L	685.4243	343.2158	668.3978	334.7025	667.4137	334.2105	6
6	803.4450	402.2262	786.4185	393.7129			L	572.3402	286.6738	555.3137	278.1605	554.3297	277.6685	5
7	917.4880	459.2476	900.4614	450.7343			N	459.2562	230.1317	442.2296	221.6185	441.2456	221.1264	4
8	1014.5407	507.7740	997.5142	499.2607			P	345.2132	173.1103	328.1867	164.5970	327.2027	164.1050	3
9	1115.5884	558.2978	1098.5619	549.7846	1097.5778	549.2926	T	248.1605	124.5839	231.1339	116.0706	230.1499	115.5786	2
10							K	147.1128	74.0600	130.0863	65.5468			1

#1-3. Peptide-centric analysis
MS/MS Fragmentation of **E.^{107}NWLYLLNPTKYPNGK^{121}.M + Deamidated (N)**
Mascot Ion Score: 51 E-value: 0.011

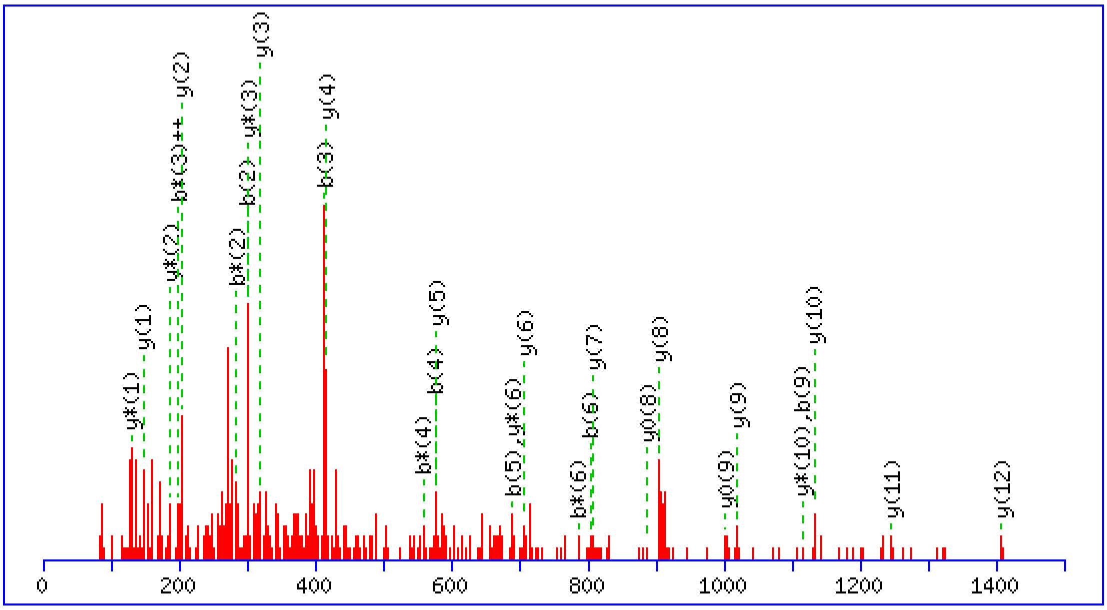

#	b	b^{++}	b*	b^{*++}	b^{0}	b^{0++}	Seq.	y	y^{++}	y*	y^{*++}	y^{0}	y^{0++}	#
1	115.0502	58.0287	98.0237	49.5155			N							15
2	**301.1295**	151.0684	284.1030	142.5551			W	1707.9105	854.4589	1690.8839	845.9456	1689.8999	845.4536	14
3	**414.2136**	207.6104	397.1870	199.0972			L	1521.8312	761.4192	1504.8046	752.9059	1503.8206	752.4139	13
4	**577.2769**	289.1421	560.2504	280.6288			Y	***1408.7471***	704.8772	1391.7205	696.3639	1390.7365	695.8719	12
5	**690.3610**	345.6841	673.3344	337.1709			L	***1245.6838***	623.3455	1228.6572	614.8322	1227.6732	614.3402	11
6	**803.4450**	402.2262	786.4185	393.7129			L	***1132.5997***	566.8035	1115.5732	558.2902	1114.5891	557.7982	10
7	917.4880	459.2476	900.4614	450.7343			N	***1019.5156***	510.2615	1002.4891	501.7482	1001.5051	501.2562	9
8	1014.5407	507.7740	997.5142	499.2607			P	***905.4727***	453.2400	888.4462	444.7267	887.4621	444.2347	8
9	**1115.5884**	558.2978	1098.5619	549.7846	1097.5778	549.2926	T	***808.4199***	404.7136	791.3934	396.2003	790.4094	395.7083	7
10	1243.6834	622.3453	1226.6568	613.8320	1225.6728	613.3400	K	***707.3723***	354.1898	690.3457	345.6765			6
11	1406.7467	703.8770	1389.7202	695.3637	1388.7361	694.8717	Y	***579.2773***	290.1423	562.2508	281.6290			5
12	1503.7995	752.4034	1486.7729	743.8901	1485.7889	743.3981	P	***416.2140***	208.6106	399.1874	200.0974			4
13	1618.8264	809.9168	1601.7999	801.4036	1600.8158	800.9116	N	***319.1612***	160.0842	302.1347	151.5710			3
14	1675.8479	838.4276	1658.8213	829.9143	1657.8373	829.4223	G	***204.1343***	102.5708	187.1077	94.0575			2
15							K	***147.1128***	74.0600	130.0863	65.5468			1

#2-1. Ion Trap GeLC-MS/MS
MS/MS Fragmentation of **M.^{168}SVGANDPFLAIFNEFK^{184}.K**
Mascot Ion Score: 74 E-value: 4.4e-06

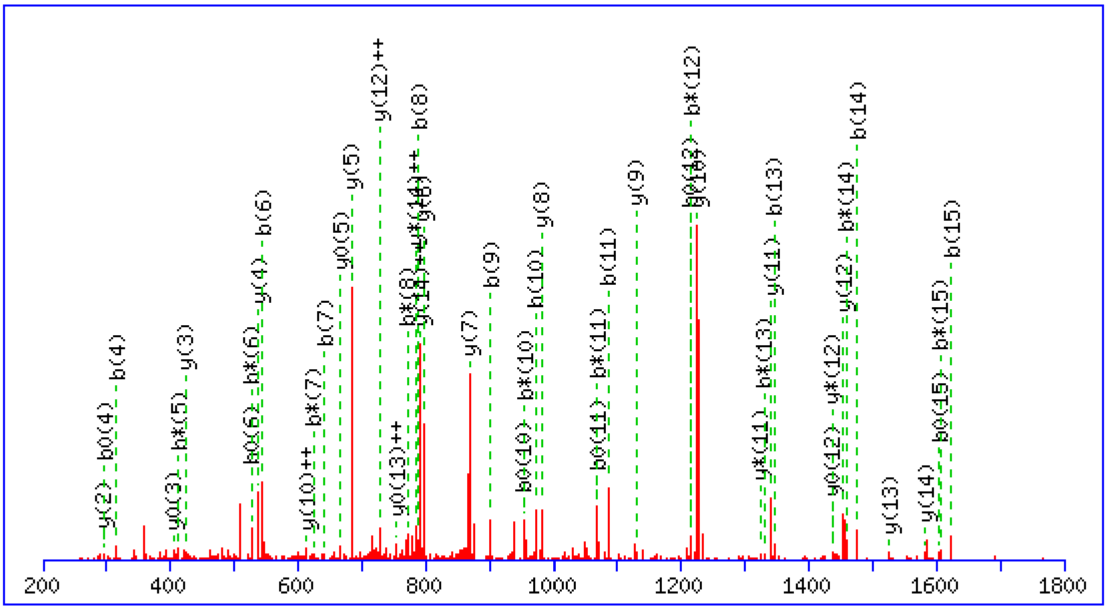

#	b	b++	b*	b*++	b0	b0++	Seq.	y	y++	y*	y*++	y0	y0++	#
1	88.0393	44.5233			70.0287	35.5180	S							16
2	187.1077	94.0575			169.0972	85.0522	V	1681.8584	841.4329	1664.8319	832.9196	1663.8479	832.4276	15
3	244.1292	122.5682			226.1186	113.5629	G	***1582.7900***	791.8986	1565.7635	783.3854	1564.7795	782.8934	14
4	**315.1663**	158.0868			297.1557	149.0815	A	***1525.7686***	763.3879	1508.7420	754.8746	1507.7580	754.3826	13
5	429.2092	215.1082	412.1827	206.5950	411.1987	206.1030	N	***1454.7314***	727.8694	1437.7049	719.3561	1436.7209	718.8641	12
6	**544.2362**	272.6217	527.2096	264.1084	526.2256	263.6164	D	***1340.6885***	670.8479	1323.6620	662.3346	1322.6780	661.8426	11
7	**641.2889**	321.1481	624.2624	312.6348	623.2784	312.1428	P	***1225.6616***	613.3344	1208.6350	604.8211	1207.6510	604.3291	10
8	**788.3573**	394.6823	771.3308	386.1690	770.3468	385.6770	F	***1128.6088***	564.8080	1111.5823	556.2948	1110.5982	555.8028	9
9	**901.4414**	451.2243	884.4149	442.7111	883.4308	442.2191	L	***981.5404***	491.2738	964.5138	482.7606	963.5298	482.2686	8
10	**972.4785**	486.7429	955.4520	478.2296	954.4680	477.7376	A	***868.4563***	434.7318	851.4298	426.2185	850.4458	425.7265	7
11	**1085.5626**	543.2849	1068.5360	534.7717	1067.5520	534.2796	I	***797.4192***	399.2132	780.3927	390.7000	779.4087	390.2080	6
12	1232.6310	616.8191	1215.6045	608.3059	1214.6204	607.8139	F	***684.3352***	342.6712	667.3086	334.1579	666.3246	333.6659	5
13	**1346.6739**	673.8406	1329.6474	665.3273	1328.6634	664.8353	N	***537.2667***	269.1370	520.2402	260.6237	519.2562	260.1317	4
14	**1475.7165**	738.3619	1458.6900	729.8486	1457.7060	729.3566	E	***423.2238***	212.1155	406.1973	203.6023	405.2132	203.1103	3
15	**1622.7849**	811.8961	1605.7584	803.3828	1604.7744	802.8908	F	***294.1812***	147.5942	277.1547	139.0810			2
16							K	147.1128	74.0600	130.0863	65.5468			1

#2-2. TX114 Q-TOF

MS/MS Fragmentation of **M.^{168}SVGANDPFLAIFNEFK^{184}.K**

Mascot Ion Score: 50 E-value: 0.0025

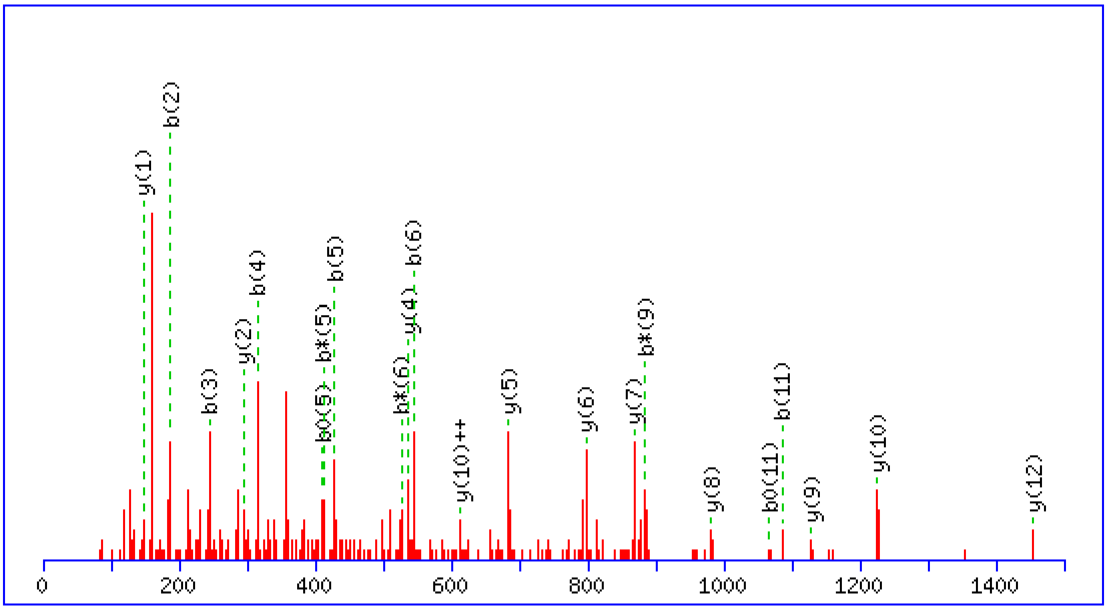

#	b	b^{++}	b*	b^{*++}	b^{0}	b^{0++}	Seq.	y	y^{++}	y*	y^{*++}	y^{0}	y^{0++}	#
1	88.0393	44.5233			70.0287	35.5180	S							16
2	***187.1077***	94.0575			169.0972	85.0522	V	1681.8584	841.4329	1664.8319	832.9196	1663.8479	832.4276	15
3	***244.1292***	122.5682			226.1186	113.5629	G	1582.7900	791.8986	1565.7635	783.3854	1564.7795	782.8934	14
4	***315.1663***	158.0868			297.1557	149.0815	A	1525.7686	763.3879	1508.7420	754.8746	1507.7580	754.3826	13
5	***429.2092***	215.1082	412.1827	206.5950	411.1987	206.1030	N	***1454.7314***	727.8694	1437.7049	719.3561	1436.7209	718.8641	12
6	***544.2362***	272.6217	527.2096	264.1084	526.2256	263.6164	D	1340.6885	670.8479	1323.6620	662.3346	1322.6780	661.8426	11
7	641.2889	321.1481	624.2624	312.6348	623.2784	312.1428	P	***1225.6616***	613.3344	1208.6350	604.8211	1207.6510	604.3291	10
8	788.3573	394.6823	771.3308	386.1690	770.3468	385.6770	F	***1128.6088***	564.8080	1111.5823	556.2948	1110.5982	555.8028	9
9	901.4414	451.2243	884.4149	442.7111	883.4308	442.2191	L	***981.5404***	491.2738	964.5138	482.7606	963.5298	482.2686	8
10	972.4785	486.7429	955.4520	478.2296	954.4680	477.7376	A	***868.4563***	434.7318	851.4298	426.2185	850.4458	425.7265	7
11	***1085.5626***	543.2849	1068.5360	534.7717	1067.5520	534.2796	I	***797.4192***	399.2132	780.3927	390.7000	779.4087	390.2080	6
12	1232.6310	616.8191	1215.6045	608.3059	1214.6204	607.8139	F	***684.3352***	342.6712	667.3086	334.1579	666.3246	333.6659	5
13	1346.6739	673.8406	1329.6474	665.3273	1328.6634	664.8353	N	***537.2667***	269.1370	520.2402	260.6237	519.2562	260.1317	4
14	1475.7165	738.3619	1458.6900	729.8486	1457.7060	729.3566	E	423.2238	212.1155	406.1973	203.6023	405.2132	203.1103	3
15	1622.7849	811.8961	1605.7584	803.3828	1604.7744	802.8908	F	***294.1812***	147.5942	277.1547	139.0810			2
16							K	***147.1128***	74.0600	130.0863	65.5468			1

#2-3. 2D gel spot
MS/MS Fragmentation of **M.^{168}SVGANDPFLAIFNEFK^{184}.K**
Mascot Ion Score: 82 E-value: 0.011

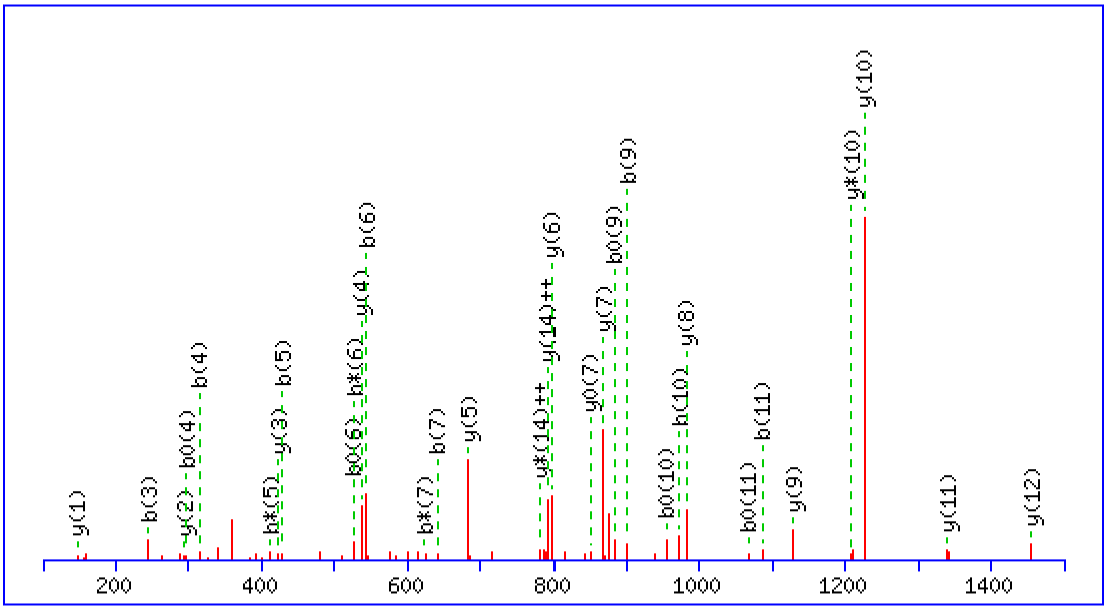

#	b	b^{++}	b*	b^{*++}	b^{0}	b^{0++}	Seq.	y	y^{++}	y*	y^{*++}	y^{0}	y^{0++}	#
1	88.0393	44.5233			70.0287	35.5180	S							16
2	187.1077	94.0575			169.0972	85.0522	V	1681.8584	841.4329	1664.8319	832.9196	1663.8479	832.4276	15
3	**244.1292**	122.5682			226.1186	113.5629	G	1582.7900	791.8986	1565.7635	783.3854	1564.7795	782.8934	14
4	**315.1663**	158.0868			297.1557	149.0815	A	1525.7686	763.3879	1508.7420	754.8746	1507.7580	754.3826	13
5	**429.2092**	215.1082	412.1827	206.5950	411.1987	206.1030	N	***1454.7314***	727.8694	1437.7049	719.3561	1436.7209	718.8641	12
6	**544.2362**	272.6217	527.2096	264.1084	526.2256	263.6164	D	***1340.6885***	670.8479	1323.6620	662.3346	1322.6780	661.8426	11
7	**641.2889**	321.1481	624.2624	312.6348	623.2784	312.1428	P	***1225.6616***	613.3344	1208.6350	604.8211	1207.6510	604.3291	10
8	788.3573	394.6823	771.3308	386.1690	770.3468	385.6770	F	***1128.6088***	564.8080	1111.5823	556.2948	1110.5982	555.8028	9
9	**901.4414**	451.2243	884.4149	442.7111	883.4308	442.2191	L	***981.5404***	491.2738	964.5138	482.7606	963.5298	482.2686	8
10	**972.4785**	486.7429	955.4520	478.2296	954.4680	477.7376	A	***868.4563***	434.7318	851.4298	426.2185	850.4458	425.7265	7
11	**1085.5626**	543.2849	1068.5360	534.7717	1067.5520	534.2796	I	***797.4192***	399.2132	780.3927	390.7000	779.4087	390.2080	6
12	1232.6310	616.8191	1215.6045	608.3059	1214.6204	607.8139	F	***684.3352***	342.6712	667.3086	334.1579	666.3246	333.6659	5
13	1346.6739	673.8406	1329.6474	665.3273	1328.6634	664.8353	N	***537.2667***	269.1370	520.2402	260.6237	519.2562	260.1317	4
14	1475.7165	738.3619	1458.6900	729.8486	1457.7060	729.3566	E	***423.2238***	212.1155	406.1973	203.6023	405.2132	203.1103	3
15	1622.7849	811.8961	1605.7584	803.3828	1604.7744	802.8908	F	***294.1812***	147.5942	277.1547	139.0810			2
16							K	***147.1128***	74.0600	130.0863	65.5468			1

#4-1. Q-TOF GeLC-MS/MS
MS/MS Fragmentation of **F.^{504}FAELNTDQEIK^{514}.E**
Mascot Ion Score: 56 E-value: 0.0024

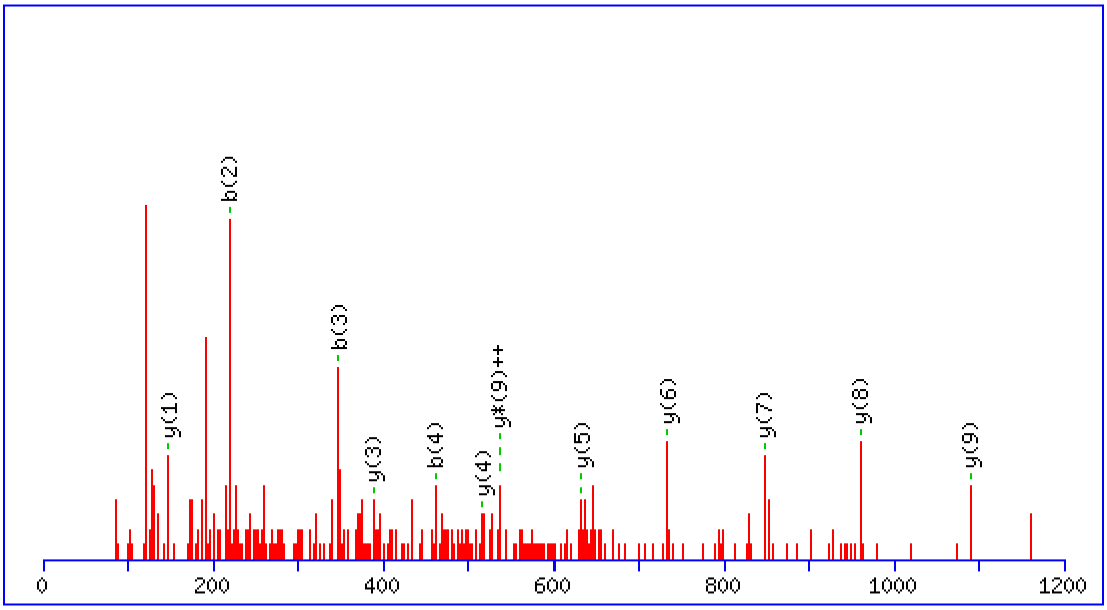

#	b	b^{++}	b*	b^{*++}	b^{0}	b^{0++}	Seq.	y	y^{++}	y*	y^{*++}	y^{0}	y^{0++}	#
1	148.0757	74.5415					F							11
2	**219.1128**	110.0600					A	1160.5794	580.7933	1143.5528	572.2800	1142.5688	571.7880	10
3	**348.1554**	174.5813			330.1448	165.5761	E	***1089.5422***	545.2748	1072.5157	536.7615	1071.5317	536.2695	9
4	**461.2395**	231.1234			443.2289	222.1181	L	***960.4997***	480.7535	943.4731	472.2402	942.4891	471.7482	8
5	575.2824	288.1448	558.2558	279.6316	557.2718	279.1395	N	***847.4156***	424.2114	830.3890	415.6982	829.4050	415.2061	7
6	676.3301	338.6687	659.3035	330.1554	658.3195	329.6634	T	***733.3727***	367.1900	716.3461	358.6767	715.3621	358.1847	6
7	791.3570	396.1821	774.3305	387.6689	773.3464	387.1769	D	***632.3250***	316.6661	615.2984	308.1529	614.3144	307.6608	5
8	919.4156	460.2114	902.3890	451.6982	901.4050	451.2061	Q	***517.2980***	259.1527	500.2715	250.6394	499.2875	250.1474	4
9	1048.4582	524.7327	1031.4316	516.2195	1030.4476	515.7274	E	***389.2395***	195.1234	372.2129	186.6101	371.2289	186.1181	3
10	1161.5422	581.2748	1144.5157	572.7615	1143.5317	572.2695	I	260.1969	130.6021	243.1703	122.0888			2
11							K	***147.1128***	74.0600	130.0863	65.5468			1

#4-2. 2D gel spot

MS/MS Fragmentation of **F.^{504}FAELNTDQEIK^{514}.E**

Mascot Ion Score: 90 E-value: 0.0014

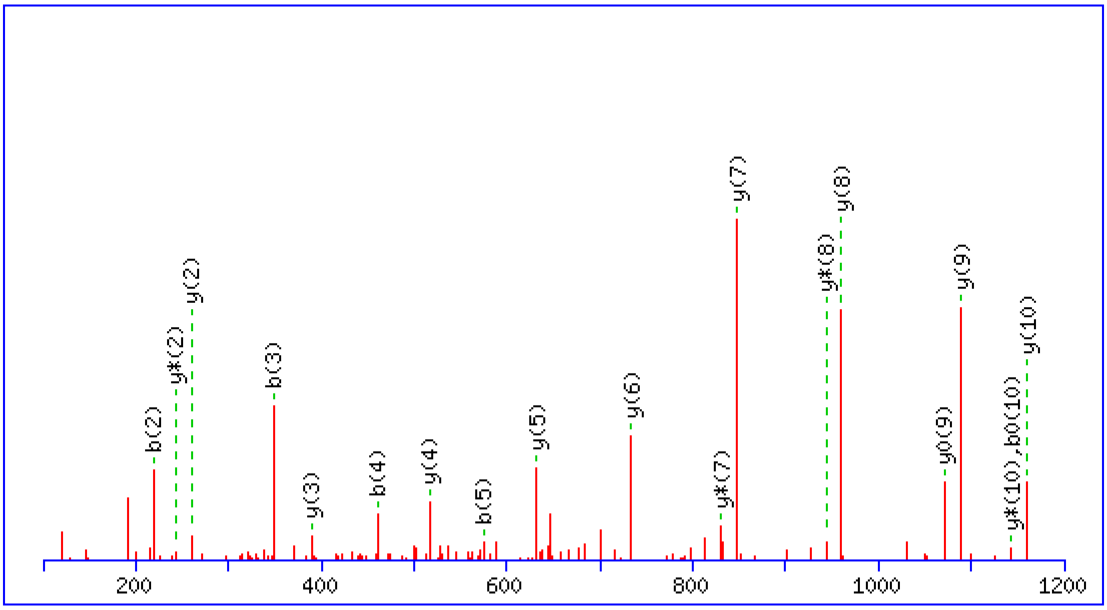

#	b	b^{++}	b*	b^{*++}	b^{0}	b^{0++}	Seq.	y	y^{++}	y*	y^{*++}	y^{0}	y^{0++}	#
1	148.0757	74.5415					F							11
2	**219.1128**	110.0600					A	***1160.5794***	580.7933	1143.5528	572.2800	1142.5688	571.7880	10
3	**348.1554**	174.5813			330.1448	165.5761	E	***1089.5422***	545.2748	1072.5157	536.7615	1071.5317	536.2695	9
4	**461.2395**	231.1234			443.2289	222.1181	L	***960.4997***	480.7535	943.4731	472.2402	942.4891	471.7482	8
5	**575.2824**	288.1448	558.2558	279.6316	557.2718	279.1395	N	***847.4156***	424.2114	830.3890	415.6982	829.4050	415.2061	7
6	676.3301	338.6687	659.3035	330.1554	658.3195	329.6634	T	***733.3727***	367.1900	716.3461	358.6767	715.3621	358.1847	6
7	791.3570	396.1821	774.3305	387.6689	773.3464	387.1769	D	***632.3250***	316.6661	615.2984	308.1529	614.3144	307.6608	5
8	919.4156	460.2114	902.3890	451.6982	901.4050	451.2061	Q	***517.2980***	259.1527	500.2715	250.6394	499.2875	250.1474	4
9	1048.4582	524.7327	1031.4316	516.2195	1030.4476	515.7274	E	***389.2395***	195.1234	372.2129	186.6101	371.2289	186.1181	3
10	1161.5422	581.2748	1144.5157	572.7615	1143.5317	572.2695	I	***260.1969***	130.6021	243.1703	122.0888			2
11							K	147.1128	74.0600	130.0863	65.5468			1

#4-3. 2D gel spot

MS/MS Fragmentation of **F.^{504}FAELNTDQEIK^{514}.E + Deamidated (NQ)**

Mascot Ion Score: 108 E-value: 2.8e-05

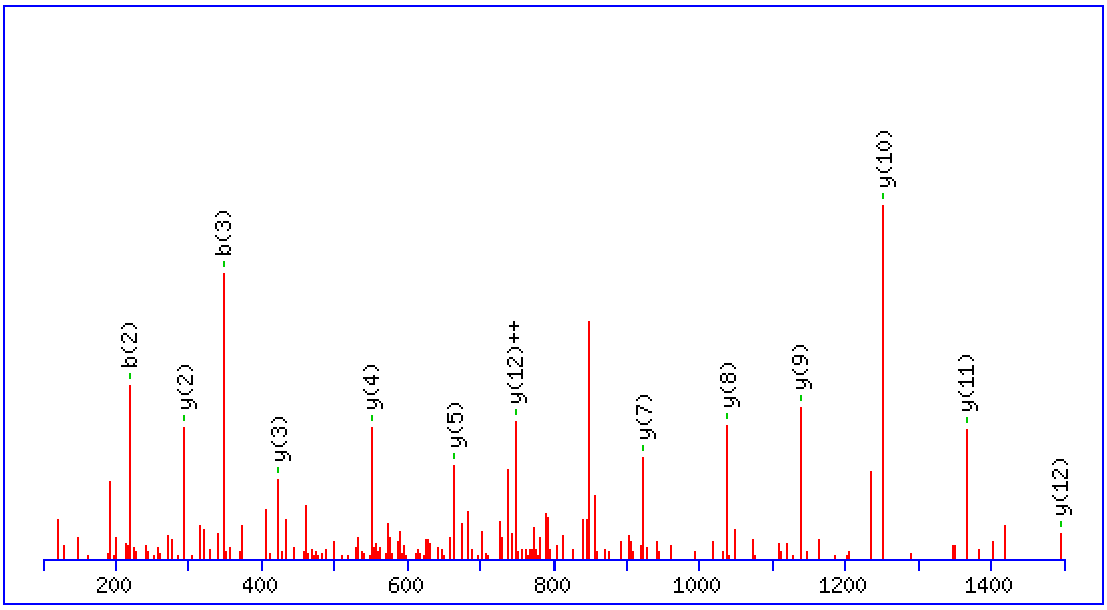

#	b	b^{++}	b*	b^{*++}	b^{0}	b^{0++}	Seq.	y	y^{++}	y*	y^{*++}	y^{0}	y^{0++}	#
1	148.0757	74.5415					F							14
2	**219.1128**	110.0600					A	1565.7693	783.3883	1548.7428	774.8750	1547.7588	774.3830	13
3	**348.1554**	174.5813			330.1448	165.5761	E	***1494.7322***	747.8698	1477.7057	739.3565	1476.7217	738.8645	12
4	461.2395	231.1234			443.2289	222.1181	L	***1365.6896***	683.3485	1348.6631	674.8352	1347.6791	674.3432	11
5	575.2824	288.1448	558.2558	279.6316	557.2718	279.1395	N	***1252.6056***	626.8064	1235.5790	618.2932	1234.5950	617.8011	10
6	676.3301	338.6687	659.3035	330.1554	658.3195	329.6634	T	***1138.5626***	569.7850	1121.5361	561.2717	1120.5521	560.7797	9
7	791.3570	396.1821	774.3305	387.6689	773.3464	387.1769	D	***1037.5150***	519.2611	1020.4884	510.7478	1019.5044	510.2558	8
8	920.3996	460.7034	903.3731	452.1902	902.3890	451.6982	Q	***922.4880***	461.7477	905.4615	453.2344	904.4775	452.7424	7
9	1049.4422	525.2247	1032.4156	516.7115	1031.4316	516.2195	E	793.4454	397.2264	776.4189	388.7131	775.4349	388.2211	6
10	1162.5263	581.7668	1145.4997	573.2535	1144.5157	572.7615	I	***664.4028***	332.7051	647.3763	324.1918	646.3923	323.6998	5
11	1290.6212	645.8143	1273.5947	637.3010	1272.6107	636.8090	K	***551.3188***	276.1630	534.2922	267.6498	533.3082	267.1577	4
12	1419.6638	710.3355	1402.6373	701.8223	1401.6533	701.3303	E	***423.2238***	212.1155	406.1973	203.6023	405.2132	203.1103	3
13	1566.7322	783.8698	1549.7057	775.3565	1548.7217	774.8645	F	***294.1812***	147.5942	277.1547	139.0810			2
14							K	147.1128	74.0600	130.0863	65.5468			1

Description of peptide-centric methods

Protein extraction and digestion for peptide fractionation by isoelectric focusing
Harvested cell pellets were freeze-dried and lyophilised cells were resuspended in 8 M urea, 100 mM NH_4HCO_3, pH 9 and sonicated with an ultrasonic probe at 80% power for 3 × 30 s on ice. Reduction and alkylation of cysteine and precipitation was carried out as described above. The protein was resuspended in 8 M urea, 100 mM NH_4HCO_3, pH 9 and digested to peptides firstly by addition of 2.5 µg of Endoproteinase LysC [Roche, Switzerland] and incubating overnight at 37°C. The sample was then diluted to 1 M urea by adding 100 mM NH_4HCO_3 prior to the addition of 2.5 µg of trypsin [Promega, USA] and incubated at 37°C for 16 h. Formic acid was added to a concentration of 1% and the peptides desalted and concentrated using an OASIS HLB SPE column [Waters, 1cc] as per manufacturer's instructions. Bound peptides were eluted with 75% acetonitrile, 0.2% formic acid. The acetonitrile was removed by lyophilisation to a volume of 50 µl and 5 µl removed for 1DLC-MS/MS using Q-TOF.
Isoelectric fractionation of peptides was performed using an Agilent 3100 OFFGEL system as per the manufacturer's instructions with the following modifications. A 13 cm pH 3-10 IPG strip [GE Healthcare] was rehydrated with a solution of 7 M urea, 2 M thiourea, 1% C7BzO and 12% glycerol for 30 min before assembling with the OFFGEL frames (12 well). 100 µg of protein digest (prepared as for SCX) was desalted and concentrated using an OASIS HLB SPE column, lyophilised to remove acetonitrile and the resulting peptide mixture was diluted to 1.8 mL with 7 M urea, 2 M thiourea, 1% C7BzO and 12% glycerol and equal volumes loaded into the 12 wells of the assembled OFFGEL frame. Isoelectric focusing was performed overnight using the following program: 150-3000 V over 3 h, 3000-10000 V over 5 h, 10000 V until 100 kVh was reached. Fractions were then recovered into separate tubes and frozen at -20°C until further analysed by 1DLC-MS/MS using Q-TOF.
